# Characterization of in vitro phenotypes of *Burkholderia pseudomallei* and *Burkholderia mallei* strains potentially associated with persistent infection in mice

**DOI:** 10.1007/s00203-016-1303-8

**Published:** 2016-10-13

**Authors:** R. C. Bernhards, C. K. Cote, K. Amemiya, D. M. Waag, C. P. Klimko, P. L. Worsham, S. L. Welkos

**Affiliations:** 1 Bacteriology Division, United States Army Medical Research Institute of Infectious Diseases (USAMRIID), 1425 Porter Street, Fort Detrick, Frederick, MD 21702-5011 USA; 2 Present Address: Edgewood Chemical Biological Centre, Aberdeen Proving Ground, Edgewood, MD 21010-5424 USA

**Keywords:** *Burkholderia*, Glanders, Melioidosis, Mouse, Spleen isolates

## Abstract

*Burkholderia pseudomallei* (*Bp*) and *Burkholderia mallei* (*Bm*), the agents of melioidosis and glanders, respectively, are Tier 1 biothreats. They infect humans and animals, causing disease ranging from acute and fatal to protracted and chronic. Chronic infections are especially challenging to treat, and the identification of in vitro phenotypic markers which signal progression from acute to persistent infection would be extremely valuable. First, a phenotyping strategy was developed employing colony morphotyping, chemical sensitivity testing, macrophage infection, and lipopolysaccharide fingerprint analyses to distinguish *Burkholderia* strains. Then mouse spleen isolates collected 3–180 days after infection were characterized phenotypically. Isolates from long-term infections often exhibited increased colony morphology differences and altered patterns of antimicrobial sensitivity and macrophage infection. Some of the *Bp* and *Bm* persistent infection isolates clearly displayed enhanced virulence in mice. Future studies will evaluate the potential role and significance of these phenotypic markers in signaling the establishment of a chronic infection.

**Electronic supplementary material:**

The online version of this article (doi:10.1007/s00203-016-1303-8) contains supplementary material, which is available to authorized users.

## Introduction

*Burkholderia pseudomallei* (*Bp*) and *Burkholderia mallei* (*Bm*), the etiologic agents of melioidosis and glanders, respectively, are classified as Tier 1 bacterial select agents. *Bm* is an obligate animal pathogen which causes a debilitating and often fatal zoonotic disease of equines. It has been eradicated in most countries, but is still found in parts of Africa, the Middle East, Eastern Europe, Asia, and South America. In contrast, *Bp* is a saprophytic, free-living organism which causes endemic infections in tropical regions such as Southeast Asia and Northern Australia. Both agents can infect humans and animals by several routes. Infections occur upon exposure to contaminated water, soil, or secretions, and through skin abrasions, inhalation, or ingestion. The diseases are manifested by protean, often nonspecific generalized symptoms such as fever and malaise, ulcerating lesions of the skin and mucus membranes, pneumonia, granulomatous abscesses in multiple organs, and septicemia. Without effective treatment, the course of these diseases may range from acute and rapidly fatal to a very protracted and frequently chronic form, as described below (Dance 2014; Gregory and Waag 2008; Welkos et al. 2015; Wiersinga et al. 2012).

These pathogens are global concerns for several reasons including the wide environmental range of *Bp*, the challenges posed in diagnosing the diseases and identifying the agents, treatment complications due to inherent and acquired antibiotic resistance, and their potential for adversarial use (Currie et al. 2000, 2010; Dance 2014; Dance et al. 1991; Fritz and Waag 2012; Naha et al. 2012, 2014; Vidyalakshmi et al. 2008; Wiersinga et al. 2012). In addition, these pathogens are potential biothreat agents because of their high aerosol infectivity and ability to cause severe disease with often nonspecific symptoms (Fritz and Waag 2012; Wiersinga et al. 2012).

These highly pathogenic *Burkholderia* species are capable of eliciting a wide range of infection states, i.e., acute, chronic, recurrent and latent (Currie et al. 2000; Nigg 1963; Tarlow and Lloyd 1971). Acute infection can manifest as a severe fulminant disease with overwhelming septicemia and pneumonia. Chronic forms are characterized as persistent infections which recrudesce clinically at varying intervals. They are characterized by signs and symptoms similar to, but milder than, those of the acute disease. These chronic infections are commonly associated with immunocompromising conditions, e.g., diabetes, and can persist for years. Reoccurring illness is also observed and can potentially be due to reinfection or relapse of a persistent infection. Latent infections are asymptomatic and can remain subclinical or alternately progress to acute melioidosis up to decades after the initial exposure. All of these forms, especially the more persistent ones, can be very challenging to diagnose and treat effectively (Dance 2014; Fritz and Waag 2012; Wiersinga et al. 2012).

To prevent the conversion of an active primary infection into a chronic or subclinical form, it is necessary to identify bacterial and host phenotypic changes which might mark infection transition to a long-term persistent state. The purpose of this study was to develop a scheme to identify in vitro bacterial phenotypes potentially associated with persistent infection and determine its capacity to distinguish isolates which persist in chronically or subclinically infected mice. These objectives are based on the hypothesis that the transition of an infection from an acute to persistent, long-term form involves adaptive changes in bacteria facilitating their resistance to antibacterial host responses. This hypothesis is supported by numerous studies on *Bp* and other Gram-negative bacteria and their ability to adapt to, or persist in, harsh environments and stressful host conditions. Previous studies documented resistance to extended periods of anaerobiosis, pH extremes, phagolysosomal contents such as antimicrobial peptides, antibiotics, and other stressors (Butt et al. 2014; Chen et al. 2014; Fauvart et al. 2011; Goodyear et al. 2012; Hamad et al. 2011; Hayden et al. 2012; Keren et al. 2004; Kint et al. 2012; Price et al. 2013; Pumpuang et al. 2011; Romero et al. 2006) and attempted to identify reliable in vitro markers for long-term colonization or chronic infection (Chantratita et al. 2007, 2012; Chen et al. 2014; Hayden et al. 2012; Tandhavanant et al. 2010; Velapatino et al. 2012). Phenotypic switching, as manifested typically by colony morphology variation, is a well-known phenomenon in the highly pathogenic *Burkholderia*. In 1924, Stanton and Fletcher first reported the presence of two colony morphotypes, a rough and mucoid form, isolated from a patient infected with *Bp* (previously *Bacillus whitmori*) (Stanton et al. 1924). Nicholls and Cantab (1930) isolated and characterized similar variants of *B*. *whitmori* from abscess material from an infected cow. The morphotypes were reversible, with the rough being more abundant and stable than the mucoid type. The colony types were associated with in vitro phenotypic differences such as production of alkaline conditions in broth, and evidence suggesting differences in virulence was described. Numerous more recent studies have supported the hypothesis that different colony morphotypes potentially reflect adaptive changes that enhance fitness in a particular environment (Austin et al. 2015; Chantratita et al. 2007; Rogul and Carr 1972; Tandhavanant et al. 2010; Velapatino et al. 2012; Wikraiphat et al. 2015; Shea, A., unpublished).

In this study, a panel of phenotyping tests for differentiating splenic mouse isolates of *Bp* and *Bm* was developed in efforts to identify markers for distinguishing isolates responsible for persistent infection. The tests included colony morphology differentiation, chemical and peptide sensitivity, macrophage infection and cytotoxicity, and LPS characterization. The goal was to identify markers which could then be further investigated to determine how *Burkholderia* transitions from an acute to chronic infection.

## Materials and methods

### Bacterial strains, media, and chemicals

The strains of *Bp* and *Bm* and their sources are described in Supplementary Table 1. *Bm* strains Turkey 1 and 10229 and *Bp* strains 316c and 4845 were obtained from the USAMRIID Therapeutics Core (TC) group. The *Bm* strain used as the prototype was *Bm* FMH, a relatively recent human clinical isolate derived from strain *Bm* ATCC 23344/China 7. This strain, *Bp* prototype strains K96243 and 1026b, and the remaining seven *Bp* strains described in the table were obtained from the USAMRIID Unified Culture Collection (UCC). All the *Burkholderia* strains were human clinical isolates with the exception of *Bp* 4845 and possibly *Bm* Turkey 1. Two of the three *Bm* strains were virulent in the hamster model of glanders (D. DeShazer, unpublished data). Additional stocks of *Bm* strain ATCC 23344/China 7 and *Bp* strain K96243 were obtained from fellow investigators [gifts from D. Waag (DW) and D. DeShazer (DD)] and used where indicated in the text. Plated media were available commercially (Thermo Fisher-Remel, Lenexa, KS) as described below, or prepared in-house. The differential/nonselective media included sheep blood agar (SBA) plates and glycerol tryptone agar (GTA). The four differential/selective media used were: OFPBL (oxidation–fermentation base polymyxin B–bacitracin–lactose) agar plates; PC/BCA (*Pseudomonas*/*Burkholderia cepacia* agar) plates with polymyxin B, ticarcillin, and dye to detect alkaline pyruvate metabolism; BCSA (*Burkholderia cepacia* selective agar) plates with polymyxin B, gentamicin, vancomycin, sucrose, and lactose with dye to detect acid production (for *Bp*); and Ashdown’s agar (AA) plates containing dyes and gentamicin (for *Bp*) or no antibiotic (for *Bm*). All were available commercially (Thermo Fisher-Remel) except GTA and AA plates which were manually prepared (Ashdown 1979; Fritz et al. 2000). Liquid growth media were glycerol tryptone broth (GTB) (Fritz et al. 2000) and cation-adjusted Mueller–Hinton broth (MHB) (BBL™, BD Diagnostics Franklin Lake, NJ). Chemicals were obtained from Sigma-Aldrich (St. Louis, MO), and antimicrobial peptides were acquired from the following sources: Sigma/Fluka, Bachem (Torrance, CA), Biopeptek (Malvern, PA), Synthetic Biomolecules (San Diego, CA), and Peptides International (Louisville, KY).

### Chemical sensitivity and enzyme production

Three platforms are used for sensitivity testing and enzyme detection: (1) GEN III MicroPlates™ (Biolog, Inc., Hayward, CA) which include 23 antimicrobial chemicals for screening sensitivity/growth inhibition; (2) agar plate assays for assessing sensitivity to hydrogen peroxide (representative of reactive oxygen species) and for detecting protease production using skim milk agar plates, as described (Sokol et al. 1979); and (3) microtiter tests to evaluate sensitivity to selected chemicals and antimicrobial peptides (AMPs) in which growth or inhibition was determined by measuring optical density (OD_630_).

The GEN III plates were inoculated as described by the manufacturer. After incubation for 48 h (*Bp*) or 72 h (*Bm*), growth was detected by measuring OD_630_. The data were scored in accordance with the Biolog instructions: Wells with growth similar to the plate positive control well were scored resistant (R), well readings less than half the positive control were sensitive (S), and readings which were borderline (±0.1 OD_630_ units below or above the R or S cutoffs) or variable were scored as R/S.

Chemicals selected for testing in the microtiter assays included NaCl (1, 4, and 8 %), nalidixic acid (5 and 50 µg/mL), surfactant Niaproof 4 (0.027 and 0.10 %), reactive oxygen species (ROS) inducer paraquat dichloride (2.5 µM), and reactive N_2_ intermediate (RNI) sodium nitrite (2 mM). The *Bm* and *Bp* strains were differentially responsive to the selected concentrations in preliminary tests; both were sensitive to 2 mM sodium nitrite as shown previously (Tandhavanant et al. 2010); and *Bp* strains were generally sensitive to 2.5 µM paraquat similarly to *Escherichia coli* (Cho et al. 2012). Twelve antimicrobial peptides (AMPs) were screened: cecropin A and P1, mastoparan 7, LL-37, magainin, histatin 5, melittin, HNP-1, hBD-2, BMAP-18, bactenecin, and CA-MA (Fox et al. 2012; Kanthawong et al. 2009; Madhongsa et al. 2013; Tandhavanant et al. 2010). Four AMPs (cecropin A, mastoparan 7, magainin, and melittin) were downselected for testing with all strains since they exhibited greatest antibacterial activity for the *Burkholderia*. Some strains were tested with an additional four because of differential sensitivity (LL-37, BMAP-18, bactenecin, and CA-MA). Due to the generally high level of resistance, a single concentration of each AMP was tested (200 µM for cecropin A and magainin and 100 µM for the rest due to reduced solubility in liquid media MHB and GTB) (Kanthawong et al. 2009; Tandhavanant et al. 2010). *E*. *coli* strains ATCC 25922 and *Bp* K96243 were used in the assays to verify activity. After addition of the antimicrobials to the trays, the wells were inoculated with strains adjusted to a concentration of approximately 1 × 10^6^ CFU/mL in MHB (*Bp*) or GTB (*Bm*). The trays were incubated and read as described for the Biolog trays, and the absorbance results were recorded as resistant (R, OD_630_ >75 % positive growth control); sensitive (S, OD_630_ <50 % positive control); borderline (R/S, OD_630_ >50 % and <75 % positive control); or very sensitive (S+, OD_630_ ≤2× the uninoculated negative control wells containing medium alone).

A separate assay for sensitivity to a combination of the enzymes lactoferrin and lysozyme (20 and 200 µg/mL, respectively), or each alone, was also conducted as described previously (Ellison and Giehl 1991; Tandhavanant et al. 2010). Microtiter plates containing PBS alone or PBS with lactoferrin, lysozyme, or lactoferrin plus lysozyme were inoculated with bacterial suspensions, and the plates were incubated for 48 h at 37 °C. The effect of the enzymes on growth was detected by measuring optical density (OD_630_) and by comparison of the densities of the treated wells to that of the untreated well (PBS alone).

### Characterization of *Burkholderia* survival and cytotoxicity in macrophages

Phagocytosis assays were developed to measure the ability of *Bp* and *Bm* to infect macrophages and to induce cell damage. The ability of the strains to replicate inside macrophages was also determined by plating for viable counts at various time points post-phagocytosis. The procedure was similar to those described previously (Arjcharoen et al. 2007; Burtnick et al. 2011; Kespichayawattana et al. 2000; Mulye et al. 2014; Tandhavanant et al. 2010; Welkos et al. 2015). The J774.A1 murine-derived macrophage-like cell line was cultured in Dulbecco’s Modified Eagle’s Medium (DMEM) with glucose, glutamine, and 10 % fetal bovine serum in 24-well plates (with or without coverslips). The cells were incubated for 2 days at 37 °C with 5 % CO_2_, at which time growth to 90–95 % confluence was achieved. On the day before the experiment, glycerol tryptone broth (GTB) was inoculated with growth from fresh SBA plate cultures of the bacteria and the flasks were incubated overnight at 37 °C with shaking at 200 rpm. Optical density measurements of the cultures were taken, suspensions adjusted to OD_620_ of 1.0 (~1×10^9^/mL) were prepared, and the macrophages were inoculated with *Burkholderia* at a target multiplicity of infection (MOI) of 5–10 bacteria per macrophage for *Bm* and <5 or 20 bacteria per macrophage for *Bp*. The infected macrophages were then incubated at 37 °C for 1 h to allow bacterial phagocytosis. The cells were washed three times to remove unphagocytosed bacteria, and fresh medium was added which contained 250–500 µg/mL of kanamycin to inactivate any residual extracellular bacteria. The cells were incubated for an additional 2 h, washed to remove the kanamycin, and then either lysed to determine the extent of phagocytosis or refed with medium containing 20 µg/mL of kanamycin. The latter cultures were then incubated for a total of 7–8 h (*Bp*) or 20–22 h (*Bm*) to determine the level of replication and cytotoxicity. The incubation times were determined based on differences in growth and infection rate. As will be described below, the shorter incubation times for *Bp* facilitated detection of variations in the responses of different strains. *Bp* strains generally grew and killed the cells more rapidly than *Bm* causing excessive cell loss when incubated for the time used in the *Bm* assays. The lysed macrophage samples were diluted and plated on SBA plates to determine quantitative viable counts of the intracellular bacteria. To account for differences in strain MOIs, the viable CFU counts were also normalized to the inoculum and expressed as percentages. In separate wells, the extent of cell cytotoxicity was measured by trypan blue (TB) dye uptake and/or by staining with propidium iodide (PI). Live cells exclude TB and PI and are unstained under phase (TB) or fluorescence (PI) microscopy, whereas dead cells are permeable and have blue (TB) or bright red (PI)-stained nuclei. Cells on coverslips were alternately stained with Diff-Quik™ histologic stain to assess macrophage condition (normal versus necrotic, apoptotic, or multi-nucleated appearance of cells and nuclei), extent of formation of multi-nucleated giant cells (MNGCs), and the relative level of residual bacterial infection. It should be noted that the nuclei in advanced-stage MNGCs are often necrotic, poorly visible, and no longer stainable by PI or TB; however, they were detectable by Diff-Quik™ stain.

Based on studies with different strains, the following phenotypes were developed for the initial screening of strains and in vivo isolates of *Burkholderia*: (1) relative bacterial survival, as determined by viable plate counts of lysed cells harvested at 6–8 h (*Bp*) or 20–22 h (*Bm*) post-phagocytosis in comparison to the concentration of bacteria present in the inoculum; (2) cytotoxicity, as assessed by live–dead staining with TB and PI to estimate the proportion of dead cells and determine the percentage of cells lost compared to the uninfected monolayer in a low power field (100× final magnification), and by staining to detect morphologic changes (Diff-Quik™); and (3) MNGC, as determined by the relative proportion of MNGCs compared to normal cells and by the nuclear morphologies (% nuclei contained in MNGCs of the total nuclei present in a higher power field [600× final magnification]). For the microscopy data, cells in a minimum of six separate fields, two for each of three Diff-Quik-stained coverslips were counted to obtain the MNGC parameters and to compare the extent of cell loss; three fields in duplicate TB- or PI-stained wells were examined to determine the proportion of dead cells and estimate of percentage cell loss. Strains selected for further analysis as a result of the screening were evaluated in assays which measured bacterial adherence and extent of uptake, as well as the time course of intracellular viability. Infected macrophage samples were lysed and diluted/plated immediately after the 1-h phagocytosis period, at the 3-h time point (after 2-h incubation in antibiotic), and after incubation of infected cells for a total of 8 h (*Bp*) or 20–22 h (*Bm*).

### LPS characterization

Differences in the gel banding profiles of LPS from different strains or spleen isolates of *Bm* and *Bp* were examined using silver-stained 10–20 % tricine gels and western blots probed with various *Bm*- and *Bp*-specific monoclonal and polyclonal antibodies, by methods described previously (Welkos et al. 2015). Silver staining was conducted using the method described by Tsai and Frasch (1982).

### Animal challenges

BALB/c mice (female, 7–10 weeks of age at time of challenge) were obtained from the National Cancer Institute, NCI/Charles River (Frederick, MD), and used in groups of 10 for challenge by aerosol or intraperitoneal (IP) routes as part of LD_50_ determinations and prospective serial collection experiments. IP challenges were conducted with various doses of *Bp* or *Bm* grown in GTB, as described previously (Welkos et al. 2015). The bacteria were quantified via OD_620_ estimations and delivered IP in 200 µL of GTB. The delivered doses were then verified by plate counts on SBA. The animals were monitored for clinical signs and symptoms for 60 days, except where indicated below. Early endpoint euthanasia was uniformly employed to limit pain and distress (Welkos et al. 2015). Aerosol challenges were done by whole-body exposure to aerosols generated by nebulization of GTB-diluted overnight cultures in a modified Henderson apparatus, as described previously (Jeddeloh et al. 2003; Waag and DeShazer 2004).

Bacterial isolates were obtained from mice surviving challenge by the aerosol or IP routes; spleens collected from mice euthanized at various times after challenge were homogenized and cultured on SBA plates for recovery of viable *Burkholderia*, as described previously (Amemiya et al. 2015).

Research was conducted under an IACUC-approved protocol in compliance with the Animal Welfare Act and other federal statutes and regulations relating to animals and experiments involving animals and adheres to the principles stated in the *Guide for the Care and Use of Laboratory Animals*, National Research Council, 2011. The facility where this research was conducted is fully accredited by the Association for Assessment and Accreditation of Laboratory Animal Care International.

### Statistical analysis

Differences in the viable counts obtained from infected macrophages were determined by *t* test or, when more than two strains were compared, by ANOVA and Tukey multi-comparison post-tests. The mean OD_630_ values determined from replicate GEN III microtiter plate experiments were evaluated for sensitivity compared to the growth control by ANOVA and multi-comparison post-tests. These statistical analyses were done with GraphPad Prism versions 5.2 and 6.0. The day 21 and 60 survival data of mice challenged by IP route with *Burkholderia* were analyzed for each strain, and the data were evaluated statistically to compare parent strain and spleen derivative virulence potencies. Bayesian probit analysis was used to calculate median lethal dose (LD_50_) and 95 % credible interval estimates and to construct dose response curves. The statistical analyses were performed using Stan 2.1.0 and R.3.1.1 software (SD Team 2013, 2014; Welkos et al. 2015).

## Results

### Characterization of a phenotyping panel for *Burkholderia* strains

The development and evaluation of assays to be used for phenotyping animal isolates were conducted using strains that were designated as “prototypes.” For *Bm*, the chosen strain was *Bm* ATCC 23344/China 7 and a derivative isolated from a relatively recent case of human glanders, FMH (Srinivasan et al. 2001 and Supplementary Table 1). Strains K96243 and 1026b were designated as the *Bp* prototypes. These strains have all been described extensively in the literature, have been sequenced, and are considered to be representative of typical virulent strains of *Burkholderia*. As a result, they are frequently employed in animal models. First, the phenotypes of the prototypes were compared to that of other strains of the same species. *Bm* strains ATCC 23344/China 7, FMH, Turkey 1, and 10229 were screened in the initial phenotyping stages. Besides the *Bp* prototype strains, *Bp* strains 316c, 4845, Bp22, and MSHR5855 were also examined initially.

#### Colony morphotyping

**Fig. 1:**
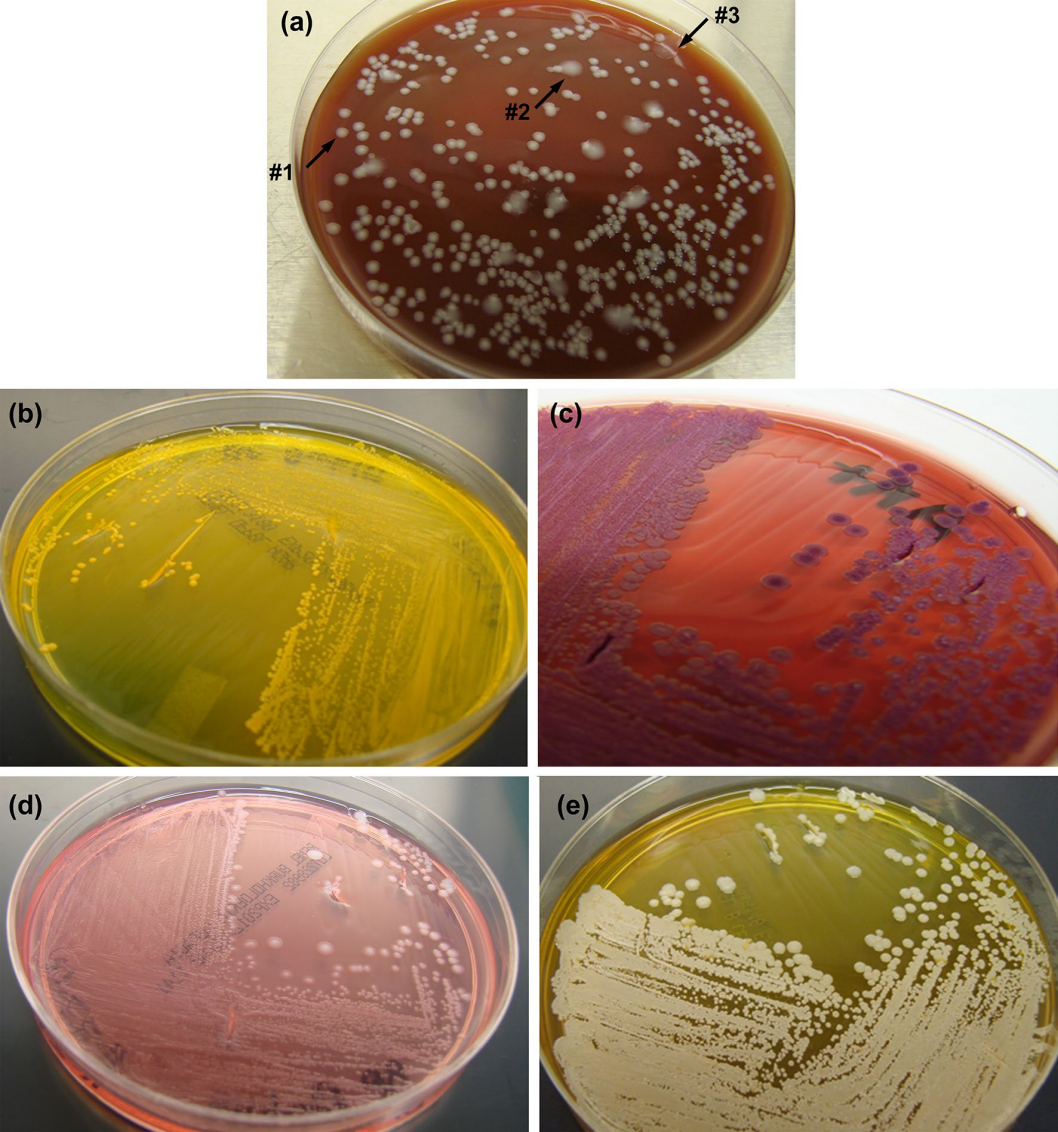
Colony morphology of *Bp* strains on selective/differential media. **a** The growth of *Bp* strain MSHR668 on SBA plates illustrates the colony morphology variation that is commonly observed for *Bp*. On this plate, three colony types are shown: the majority of the colonies are small, round, and smooth (*#1*); the next most numerous are larger, shinier, and less opaque, and have a translucent edge (*#2*); and there are two “water splat” colonies that are large, flat, and translucent/clear, and have an irregular edge (*#3*). The growth of *Bp* strain K96243 illustrates morphologies produced by *Bp* on several selective media (**b**–**e**). **b** OFPBL plate. **c** AA plate showing a common colony morphology of *Bp* strains. **d** BCA plate. Strains metabolizing the peptones and pyruvate produce alkaline conditions changing the agar indicator color from orange to pink. **e** BCSA plate, used for *Bp* only. This medium distinguishes strains which metabolize the peptones (alkaline color change) or catabolize the carbohydrates (acidic color change from deep pink to yellow, as shown for K96243)

To characterize colony morphology of *Bp* and *Bm*, six selective and/or differential media were used: SBA, GTA, AA, OFPBL, BCA, and BCSA. In addition, skim milk agar was included to assay for protease production. Because *Bm* strains are susceptible to gentamicin, a modified version of Ashdown’s medium lacking the antibiotic was used for *Bm* strains. The use of six media allowed strains to be differentiated morphologically in at least seven characteristics, i.e., colony size, color, moistness, opacity, circumference shape, surface texture, and changes in agar color (the extent of hemolysis activity on SBA plates or change in indicator color due to bacterial metabolic activity, e.g., acid production). Colony phenotyping for *Bp* strains is illustrated in Fig. 1. Whereas a variety of different randomly occurring phenotypes were readily distinguished among the *Bp* strains, the strains of *Bm* exhibited few morphological differences (data not shown). The variable production of different colony morphotypes often observed for *Bp* strains was especially apparent using SBA, GTA, and AA. The variant colonies demonstrated on the SBA plate (Fig. 1a) are those of *Bp* strain MSHR668; strain K96243 was used to illustrate growth by *Bp* on OFPBL, AA, BCA and BCSA selective media, respectively (Fig. 1b–e). Despite the random morphotypic heterogeneity often observed in *Bp* strains, the flat, rough colonies which appear in the majority on the AA plate (Fig. 1c) illustrated one of the morphotypes commonly observed on AA plates among the *Bp* strains studied here. Specifically, this morphotype was a relatively flat, rough, often dry, and striated colony type, which was observed for many strains, albeit in varying relative abundance. Similar morphotypes were among those described previously by Chantratita et al. (2007) in a set of seven morphotypes (I–VII). The colonies we observed in the *Bp* parent strains varied in color and central colony texture but resembled variants of Morphotype I (rough colony center) or IV (striated, with smooth umbonated center), and had a purple color ranging from light to dark, as described for Morphotypes I or IV and Morphotype II, respectively. The colonies exhibited differences between plating on AA in color, moisture, and surface texture, as illustrated by the 406e parent strain in Supplementary Table 5A and B. However, this flat, rough colony type and variants thereof may represent the major colony phenotype isolated from patients with melioidosis and the colony from which switching to other colony morphotypes occurred in vivo, as reported by Chantratita et al. (2007). In addition to AA plates, this morphotype was characterized by relatively flat, nonmucoid colonies on SBA plates, as described previously (Wikraiphat et al. 2015), and by acid production on OPFBL plates. A form similar to this flat, rough morphotype was produced in varying proportions by all of the six strains described below in the mouse isolate studies (K96243, 1026b, 1106a, 406e, MSHR305, and HBPUB10134a) and by Bp22, MSHR5855, 316c, and 4845.

#### Chemical sensitivity testing and enzyme production

Chemical sensitivity tests in the Biolog GEN III panel were evaluated as a candidate phenotyping tool. Several strains of *Bm* and *Bp* were used to determine optimal incubation times, assess the range of responses, and establish the scoring. The responses of prototype strains *Bm* ATCC 23344/China 7 and *Bp* K96243 were compared, and the results indicated that *Bp* is potentially more resistant to potential environmental, intra-phagocyte, and antimicrobial stresses than *Bm* (Supplementary Table 2). *Bm* and *Bp* were similarly resistant to some antibiotics (rifampin, lincomycin, and vancomycin), and *Bp* was more resistant than *Bm* to other antibiotics (troleandomycin, nalidixic acid, and minocycline). Both species exhibited a similar sensitivity to NaCl concentrations (variable growth in 1 % but not 4 or 8 %) and were fully resistant to mild pH stress (pH 6). *Bm* was moderately resistant, while *Bp* was fully resistant to low pH stress (pH 5). *Bp* K96243 was more resistant to potassium tellurite and to the surfactant Niaproof 4 than *Bm* ATCC 23344/China 7.

Further studies were done with additional *Burkholderia* strains. *Bm* strain ATCC 23344/China 7 did not show any differences associated with the source of the strain (different culture collections of the original or human isolate FMH), and there were few variations in sensitivity between *Bm* strain 23344 and the other two strains tested (*Bm* Turkey 1 and 10229) (data not shown). In assays with 10 strains of *Bp* (K96243, 1026b, 316c, 4845, MSHR668, 1106a, 406e, HBPUB10134a, MSHR305, and Bp22), several phenotypes exhibited strain-specific differences in sensitivity, to include the antibiotics nalidixic acid, minocycline, and aztreonam, as well as Niaproof 4 and potassium tellurite (Table 1). Inter-experimental variability in sensitivities to aztreonam, potassium tellurite, and Niaproof 4 was noted for some of the *Bp* strains and to a lesser extent for *Bm* strains. As described below, potentially confounding effects of this variability were alleviated in all phenotypic comparisons of in vivo isolates with the challenge strain by the inclusion of the parent strain in all in vitro assays.

**Table 1:** Comparison of chemical sensitivities of different *B*. *pseudomallei* strains: Biolog phenotyping panel

Chemical	K96243/DW		K96243/DD		1026b		316c		4845	
	Mean	SD	Mean	SD	Mean	SD	Mean	SD	Mean	SD
**Pos. ctrl.**	1.644	0.141	1.558	0.019	1.706	0.064	1.719	0.140	1.567	0.256
1 % NaCl	1.279	0.153	1.492	0.015	1.540	0.138	1.673	0.002	1.380	0.016
Sodium lactate	1.841	0.167	1.737	0.027	1.820	0.082	1.826	0.025	1.669	0.191
Troleandomycin	1.356	0.309	1.280	0.068	1.406	0.296	1.332	0.511	1.253	0.443
Lincomycin	1.636	0.145	1.735	0.027	1.824	0.071	1.912	0.018	1.783	0.058
Vancomycin	1.730	0.181	1.692	0.083	1.826	0.077	1.795	0.134	1.729	0.063
Nalidixic acid	1.204	0.233	**−0.097**	0.009	**−0.059**	0.053	1.679	0.146	1.249	0.188
Aztreonam	**0.097**	0.040	***0.647***	0.312	**0.237**	0.106	***0.318***	***0.170***	***0.253***	0.332
pH 6	1.734	0.109	1.658	0.074	1.774	0.069	1.807	0.102	1.671	0.162
4 % NaCl	**−0.063**	0.066	**−0.121**	0.010	**0.026**	0.089	**0.052**	0.218	**−0.052**	0.148
Fusidic acid	**0.024**	0.063	**−0.117**	0.000	**−0.088**	0.038	**0.224**	0.212	**−0.060**	0.121
Rifampin	1.667	0.187	1.617	0.121	1.809	0.042	1.847	0.188	1.725	0.173
Guanidine HCl	**0.155**	0.056	**0.187**	0.007	**0.242**	0.104	**0.337**	0.106	**0.294**	0.049
Tetrazolium violet	2.908	0.022	2.919	0.025	2.892	0.021	2.955	0.027	2.859	0.008
LiCl	**−0.022**	0.151	**−0.096**	0.004	**0.006**	0.123	**0.062**	0.120	**−0.038**	0.142
Sodium butyrate	**0.074**	0.119	**−0.098**	0.002	**0.050**	0.096	**0.197**	0.145	**−0.021**	0.060
pH 5	1.828	0.152	1.769	0.066	1.837	0.062	1.692	0.254	1.797	0.126
8 % NaCl	**−0.141**	0.015	**−0.009**	0.141	**−0.113**	0.024	**−0.116**	0.011	**−0.149**	0.007
d-Serine	**−0.072**	0.028	**−0.102**	0.002	**0.236**	0.155	**0.221**	0.025	**0.063**	0.057
Minocycline	1.267	0.252	**−0.098**	0.012	**−0.074**	0.020	1.695	0.117	1.377	0.107
Niaproof 4	**0.481**	0.073	**0.589**	0.005	**0.153**	0.293	0.949	0.325	***0.516***	0.550
Tetrazolium blue	3.023	0.049	2.983	0.104	2.980	0.081	3.056	0.069	2.996	0.003
Potassium tellurite	***0.877***	0.971	1.605	0.010	1.002	0.970	***1.291***	0.787	1.645	0.046
Sodium bromate	**−0.121**	0.017	**−0.102**	0.011	**−0.101**	0.016	**−0.106**	0.015	**−0.136**	0.008

The criteria for sensitivity are similar to those defined by Biolog, Inc.

Bold = growth densities (OD_630_) less than half that of positive control well are sensitive

Unchanged = growth densities similar to positive control are resistant

Bold italics = density compared to positive control is borderline or variable (different experiments or strain sources)

In addition to the Biolog panel, *Bm* and *Bp* prototype strains were assayed independently for sensitivity to selected chemicals. Initial findings with *Bm* FMH and *Bp* K96243 indicated that both were sensitive to 2 mM sodium nitrite (RNI), as shown previously (Tandhavanant et al. 2010). Also, *Bm* was more sensitive than *Bp* to H_2_O_2_, and both were similar in sensitivities to NaCl and Niaproof 4; *Bp* K96243 was more sensitive to these two chemicals in the individual assays than in the Biolog plates (Table 1, parent strains in Table 2 and Supplementary Tables 2 and 3, and data not shown). The concentrations of chemicals used in the Biolog microplate assays are proprietary and results can only be qualitatively compared to assays which use defined concentrations (B. Bochner, personal commun.). Responses of *Bm* and *Bp* were variable to 5 µg/mL nalidixic acid, but *Bm* strains were generally sensitive and *Bp* strains were generally resistant (parent strains in Table 2, Supplementary Tables 3 and 4, and data not shown). All strains were sensitive to 50 µg/mL nalidixic acid. Thus, the chemical sensitivity data indicated that *Bp* appeared to be generally more resistant to potential environmental and host antimicrobial stresses than *Bm*. Due to evidence of strain variability, the subsequent analyses of phenotypic changes in strains recovered during infection required that responses of the original parent strain be tested in each assay. The direct comparison of parent and in vivo isolates responses allowed relative differences to be detected.

**Table 2:** In vitro phenotype screening of in vivo isolates of *B*. *mallei*: representative results

Strain^a^	Biolog^b^		Individual chemical sensitivities^c^								Antimicrobial peptides^c^				
	No. resistant/total (23)	Change	H_2_O_2_: 0.02, 0.1 %	NaCl: 1, 4 %	Nalidixic acid: 5, 50 µg/mL	Niaproof 4: 0.027, 0.10 %	RNI: 2 mM	Cecropin P1	Cecropin A	LL-37	Mastoparan 7	Magainin	Melittin	Histatin 5	Proteolytic activity (mm)
Spleen isolates: Bm FMH aerosols															
*Bm* FMH parent	9^d^	none	4.5, 4.5	S, S+	S, S+	S+, S+	S	R	R/S	R	R/S	R/S	S	R	1.5
			4, 5	S, S+	S/R, S+	S+, S+	S	S	nd	S	R	S	S	nd	nd
26-2	10	**R**-nal. acid	NC^e^	S, S+	**R**, S	S+, S+	S	nd^e^	R−	nd	S+	**R**	**R**	nd	2.5–3
26-3	10	**R**-nal. acid	4.0, 7	S, S+	**R**, S	S, S+	S	nd	R−	nd	S+	**R**	**R**	nd	2.5
*Bm* FMH parent	9^d^		4, 5	S, S+	R/S, S+	S+, S+	S	nd	S	R	S	S	nd	nd	
45-7	9, diffs	**R**-nal.A; ***S***-linco	NC	NC	**R**/S+	**S**, S+	NC	nd	NC	NC	***S*** **+**	NC	NC	nd	nd
45-9	9, diffs	**R/S**-nal.A; ***S***-Nacl	NC	NC	NC	NC	NC	NC	nd	NC	**R**	NC	NC	nd	nd
*Bm* FMH parent	9^d^	–	4.5, 6	S+, S+	S, S+	S+, S+	S+	nd	S	nd	S+	S	S	nd	nd
60-1	NC	None	4.5, 6	S+, S+	S, S+	S+, S+	S+	nd	**R**	nd	S	S	**R**	nd	“
60-3	NC	None	5, 5(haze)	S+, S+	**R**, S	S+, S+	S	nd	S	nd	S	**R**	**R**	nd	“
60-3	NC	None	4.5, 5(haze)	S, S+	**R**, S	S+, S+	S	nd	**R**	nd	S	**R**	**R**	nd	“

^a^Isolates are from spleens of mice infected with *Bm* FMH. With the exception of the infecting strain (parent), the strains are identified by the day post-inoculation on which spleens were collected from the infected mice and by the isolate number. Responses of the isolates which varied from the parent are bolded (more R) or in bold italics (more S)

^b^Sensitivities were determined with the GEN III panel of 23 chemicals (Biolog). The Biolog criteria were used for resistance (R, >50 % positive control OD_630_), sensitive (S, <50 % pos. control), and borderline or variable (R/S). “Diffs” indicates that the strain was resistant to the same total no. chemicals as the parent but differed in specific chemical sensitivities

^c^Sensitivities to specific chemicals and antimicrobial peptides are determined by OD_630_ readings: values >75 % positive control (resistant, R), >50 % and <75 % pos. ctrl (borderline, R/S), <50 % (sensitive, S), and <×2 negative control (highly sensitive, S+). For the H_2_O_2_ plates, the values (mm) are the zones of cleared growth proximal to the H_2_O_2_ spot

^d^In Biolog experiments, *Bm* strain FMH was resistant to nine conditions: 1 % NaCl, Na lactate, pH 5 and 6, tetrazolium blue and purple, vancomycin, rifampin, and lincomycin. It was variable but usually sensitive to nalidixic acid in the Biolog and individual assays (low, 5 µg/mL; and high, 50 µg/mL). *Bm* FMH was generally sensitive to 13 conditions: NaCl (4 % and 8 %), fusidic acid, d-serine, guanidine HCl, Niaproof 4, LiCl, potassium tellurite, aztreonam, Na butyrate, nalidixic acid, Na bromate, and troleandomycin

^e^NC = same as parent (no change); nd = not done

The *Burkholderia* have been reported to be notably resistant to most AMPs at concentrations ≤200 µM. Although *Bp* has been shown to be sensitive in vitro to the peptide LL-37 (Kanthawong et al. 2009; Tandhavanant et al. 2010), we observed resistant to borderline resistant responses to LL-37 in repeated tests with *Bm* FMH and most *Bp* strains. These findings are exemplified for the parent strain data in Table 2 and Supplementary Tables 3 and 4. Sensitivities to five more AMPs were initially evaluated with the prototype strains. *Bm* and *Bp* were resistant to histatin 5, *Bp* was resistant more often than *Bm* to cecropin A and melittin, and both were often relatively sensitive to mastoparan 7 (data not shown). In skim milk agar assays for protease production, prototype strain *Bp* K96243 produced larger zones of clearing than *Bm* 23344/China 7, possibly due to the secretion of more protease by *Bp* and/or its faster rate of growth (data not shown). Since the *Burkholderia* demonstrated some variability in AMP sensitivity between experiments, the impact of this on later analyses of the sensitivities of infected mice isolates was mitigated by direct comparisons to the activity of the parent strain in each assay.

#### Macrophage phenotypes of the *Bm* and *Bp* strains

**Fig. 2:**
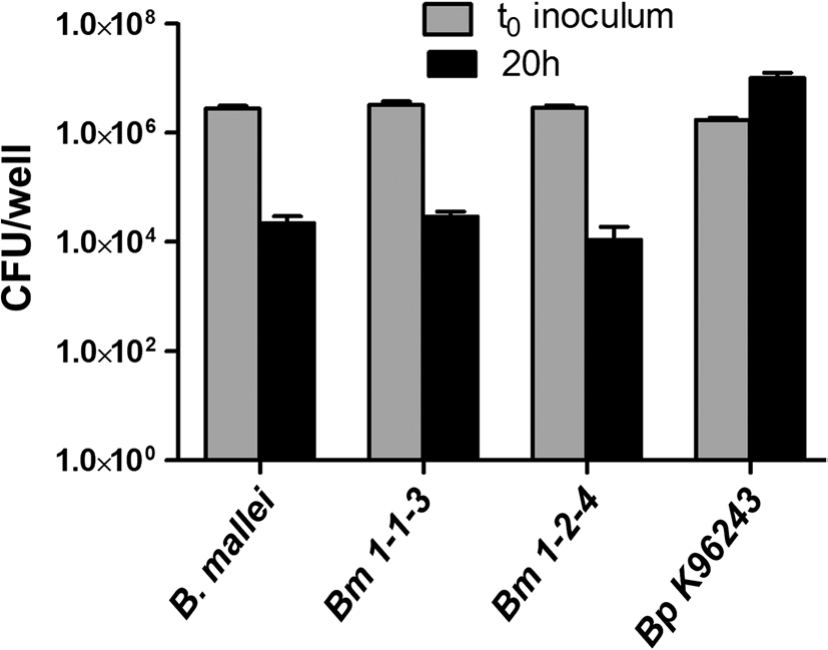
Comparison of macrophage survival of *Bp* and *Bm* strains. The J774.A1 macrophage cell line was infected with *Bp* strain K96243 at a MOI of 4.3; or with *Bm* strain FMH or its spleen isolates *Bm* 1-1-3 or *Bm* 1-2-4 at MOIs of 6.8, 7.95, and 7.1, respectively. The *paired bars* correspond to the mean viable counts from triplicate wells determined at t_0_ (macrophage inocula) and after 20-h incubation of infected macrophages (t_20_). Spleen isolates *Bm* 1-1-3 and *Bm* 1-2-4 were collected from IP-infected mice on days 3 and 16, respectively

The J774.A1 cell line was used to develop several macrophage phenotypes for comparing different strains of *Bm* and *Bp*. Several parameters were identified including relative bacterial survival over time within the macrophage and several infection-induced cytotoxic effects involving cell killing and MNGC production. In screening assays, the number of viable *Bm* recovered was much less than the number in the inoculum. In contrast, *Bp* prototype strain K96243 proliferated and exhibited a large increase in CFU compared to the input concentration, i.e., 600-fold after 20 h (Fig. 2), but also after incubation for much shorter times, as described below. As shown in Fig. 2, for the *Bm* strains, there was a large decline in viable counts recovered 20 h after infection (126-, 112-, and 267-fold, respectively, for FMH parent and isolates 1-1-3 and 1-2-4 from infected spleens). At t_20_, for all three *Bm* strains, <1 % of the input bacterial inoculum could be recovered (0.8, 0.9, and 0.4 %, respectively). In contrast, the *Bp* strain K96243 exhibited a 5.8-fold (581 %) increase in number of CFU/well at t_20_ compared to the initial concentration added at t_0_. Despite the lower MOI of the K96243 inoculum (*p* < 0.8 × 10^−5^), the number of viable bacteria recovered at 20 h was significantly greater than the viable organisms recovered of the *Bm* strains (*p* < 0.001 each, by ANOVA and Tukey post-test paired comparisons). This pattern of survival and proliferation in macrophages of K96243 was also observed for other *Bp* strains from diverse sources (Supplementary Table 1) (Welkos et al. 2015; data not shown). Furthermore, both *Bm* FMH and *Bp* K96243 induced MNGC formation, yet *Bp* was significantly more cytotoxic and the infection resulted in ≥90 % killing and loss of adherent cells from the monolayer. However, *Bm* cytotoxicity was observed to differ significantly in association with the MOI and was generally less for MOIs <5 and more for MOIs >5 in our model system. In contrast, the cytotoxicity associated with strains of *Bp* was less affected by the MOI (discussed further below). The more extensive cytotoxicity demonstrated for K96243 was also observed for other *Bp* strains from diverse sources (Supplementary Table 1) such as 1026b, Bp22, and MSHR5848 (Welkos et al. 2015; data not shown).

### Analysis of in vivo isolates of *Burkholderia*

The phenotypic characterization strategy was employed to examine in vivo isolates of *Bm* and *Bp* for changes which might be associated with progression of an acute infection to a more persistent form. Isolates from spleens collected from 3 to 180 days post-challenge from mice involved in aerosol or IP LD_50_ studies were analyzed. Spleen isolates obtained from infections by either route were generally observed to be similar in their phenotypic comparisons to the corresponding parent challenge strain. Because of the in vitro variability of the pathogenic *Burkholderia* spp., the original challenge strain was included in all in vitro assays for comparison with the spleen isolates. To categorize the in vivo isolates, we empirically defined three phases of infection: an acute phase (≤21 days post-challenge), a midterm phase (21–59 days), and a long-term phase (≥60 days).

#### Comparison of isolates and parent strains by colony morphology phenotyping

Bacteria isolated from mice euthanized during the acute phase of infection with either *Bm* or *Bp* did not differ significantly from the parent challenge strain in their colony morphotypes (data not shown). To confirm this observation, mice were infected IP with 5 LD_50_ doses of either strain 1106a or HBPUB10134a and survivors were serial sampled on days 3, 7, or 14 post-infection for spleen colony isolations. These two strains represent mice that were, respectively, the least and most virulent by the intraperitoneal route (Welkos et al. 2015). For both strains, the spleen isolates exhibited no significant differences from the challenge strain, as typically observed for early stage isolates. Similar findings were observed for isolates obtained on days two through six after aerosol challenge with HBPUB10134a (data not shown).

For spleen isolates from aerosol challenges with the *Bm* FMH strain, there was an apparent increase in the overall number of morphotypic differences between spleen isolates and parent with longer times post-challenge. For example, most *Bm* FMH isolates collected ≤day 45 varied little from the parent challenge strain, while isolates collected 119 days after challenge were larger on SBAP, OFPBL, and BCA plates (data not shown). Similarly, for some *Bp* strains there was an increase in the overall number of small differences in colony morphology with time post-challenge for isolates. For example, isolates of *Bp* strain 1106a collected at times later than day 49 exhibited numerous alterations compared to the parent in colony size (several media) and morphology (e.g., opacity and moistness). For three *Bp* strains (1106a, MSHR668, and 406e), there was a reduced incidence of multiple colony variants on SBA, GTA, and/or AA displayed by isolates; i.e., whereas the parent exhibited two distinct variants differing in several characteristics, only one of the variant types was observed in all the isolates. This is demonstrated for strain 406e in Supplementary Table 5A and B. For some of the strains, such as MSHR305 and HBPUB10134a, there was a paucity of mice surviving at later time points, and among the survivors, few if any had residually infected spleens. However, isolates obtained 33 and 35 days post-infection with MSHR305 were larger on both SBAP and AA (day 33) or on SBAP (day 35), and half of those tested had enhanced metabolic activity on BCA plates, as indicated by color change in this medium illustrated in Fig. 1d (data not shown). Thus, it can be concluded that *Bp* isolates routinely exhibited differences in colony morphology from the parent strain, yet an overall common pattern of change in isolate colony morphotype was not observed among the *Bm*/*Bp* strains examined. Therefore, we considered the presence of any distinct difference in colony morphotype compared to parent colony morphology to be important as one of the bases of selecting isolates for subsequent phenotypic analyses. Colony morphology differences together with GEN III plate differences (described below) were used as criteria for selecting strains for subsequent, more complex phenotype assays.

#### Comparison of isolates and parent strains in antimicrobial sensitivities

Spleen isolates were initially screened for sensitivity to 23 antimicrobial chemicals using GEN III plates, and selected isolates were then tested in the additional antimicrobial sensitivity assays. All assays included the corresponding parental challenge strain for intra-experimental comparisons. Isolates obtained from spleens collected between 3 and 16 days after challenge did not differ significantly in sensitivity from the parent *Bm* FMH or *Bp* strain. Such isolates included, for example, those from spleens collected 3 and 16 days after IP challenge with *Bm* FMH (Fig. 2), strains from samples obtained 2–14 days after IP injection of* Bp* strains 1106a or HBPUB10134a, and spleens collected 2–6 days after aerosol and/or IP exposure to HBPUB10134a or 1026b, as described above.

Results with spleen isolates acquired from survivors on or after day 26 after aerosol exposure to *Bm* FMH are illustrated in Table 2. These isolates often differed in sensitivity, typically being more resistant than the parent to several antimicrobials, especially nalidixic acid and the AMPs cecropin A, magainin, and melittin. Isolates from the spleens of mice infected with the seven *Bp* strains were obtained from 17 experiments (12 aerosol challenges of 7 strains and 5 IP challenges of 5 strains). The results of chemical sensitivity screening of the isolates are summarized in Table 3 and Supplementary Table 6. The latter details the sensitivity differences between strains for the chemicals in Table 3. The data for strain HBPUB10134a are not included in these tables since isolates from the acute stage of infection were only available (≤day 14). The changes in chemical sensitivities of the in vivo isolates which are described in Table 3 and Supplementary Table 6 can be summarized as follows. Differences in sensitivities of spleen isolates compared to the *Bp* parent strain were observed most frequently for two antibiotics, nalidixic acid and minocycline, for which isolates from five or three of six strains tested, respectively, differed in sensitivity from the parent strain. Isolates with enhanced resistance to nalidixic acid were recovered for all five of the strains. Isolates with altered sensitivities compared to the *Bp* parent strain were detected most commonly for four of the chemicals tested (Niaproof 4, nitrite, NaCl, and potassium tellurite) and the AMPs mastoparan 7, BMAP-18, and magainin. As shown in Table 3, overall, these *Bp* isolates most often exhibited an increased resistance to the antimicrobial substances compared to the parent. For example, isolates with greater resistance to Niaproof 4 were recovered for five of the six *Bp* strains evaluated and isolates with greater resistance to NaCl were detected for all three *Bp* strains from which bacteria with altered NaCl sensitivities were obtained. These findings can be further illustrated with the isolate sensitivity results of strains K96243 and 1026b. Supplementary Table 3 describes sensitivity patterns of K96243 isolates obtained on day 70 after aerosol exposure. Despite parental strain variability, these isolates were generally enhanced in their resistance to potassium tellurite, Niaproof 4, and several AMPs (mastoparan 7 and potentially LL-37, melittin, and BMAP-18) but more sensitive to fusidic acid in the GEN III assays. The K96243 isolates were sensitive to mastoparan 7 at day 28 and resistant at days 49 and 70 (Supplementary Table 3 and data not shown), suggesting a time-associated response. The responses of the day 30 and day 60 isolates from challenge experiments with *Bp* strain 1026b are shown in Supplementary Table 4. The data suggested an increase in the resistances of 1026b isolates with time post-challenge as seen with nalidixic acid and various AMPs such as bactenecin and BMAP-18. These findings were intriguing though additional serial kill studies will be required to confirm a time-associated pattern of change among other *Bp* strains. It will be necessary to evaluate many isolates at each of the time points post-challenge from mice. There was no apparent time-associated pattern in antimicrobial resistances of the in vivo isolates of strain *Bm* FMH (Table 2).

**Table 3:** Summary of differences between parent *B*. *pseudomallei* and in vivo isolates in chemical sensitivities

Chemical	No. strains with: variant isolates/total no. strains^a^	Increased in	
		Sensitivity	Resistance
Niaproof 4	6/6	3^b,c^	5^b,c^
Nalidixic acid	5/6	1	5^b^
Mastoparan 7	4/6	1	4^b^
BMAP-18	3/4^e^	0	3
Minocycline	3/6	2	2^b^
Nitrite	3/6	2	1
NaCl	3/6	0	3
Potassium tellurite	3/6	1^b^	3^b^
Magainin	2/6	0	2
Melittin	2/6	1	1
d-Serine	2/6	1	1
Others	1/6^d,e^	–	–

^a^Spleen isolates from aerosol or IP challenge experiments of seven *Bp* strains were obtained (17 total experiments). The variant responses of the isolates consisted of significant increases in either sensitivity (S) or resistance (R) to the antimicrobial chemical compared to the challenge strain, as described in the methods. The data do not include isolates exhibiting no differences from the parent. For strain HBPUB10134a, no isolates were obtained from survivor spleens cultured later than 14 days after challenge, so the data are not included in the table

^b^The sensitivity of isolates collected at early and later time points differed (nalidixic acid, potassium tellurite, Niaproof 4, and mastoparan 7) or individual isolates from a given challenge experiment collection responded differently, being either more sensitive or more resistant than the parent (minocycline, Niaproof 4, potassium tellurite, and mastoparan 7) as described in the text

^c^Different responses by the same isolate were observed in Biolog and antimicrobial sensitivity microtiter assays, but chemical concentrations in the Biolog system are proprietary and unavailable

^d^For one *Bp* strain each, the sensitivity to the chemical of an isolate(s) varied from that of the parent as follows: aztreonam (R), guanidine HCl (R), LL-37 (R), CA-MA (R), and bactenecin (variable)

^e^Peptides BMAP-18, CA-MA, bactenecin, and LL-37 were tested in four of the six strains only

#### Comparison of isolates and parent strains in macrophage phenotypes

To prospectively evaluate macrophage virulence during the acute phase of infection, mice were intraperitoneally challenged with *Bp* 1106a and serial killed on days 3, 7, and 14 post-infection, as described above. The 1106a spleen isolates exhibited a progressive change in macrophage virulence. The day 3 isolate was similar in extent of phagocytosis, survival, and cytotoxicity to the parent, and the day 7 isolate was phagocytosed to a greater extent but otherwise similar in macrophage parameters (data not shown). However, the day 14 isolate of 1106a (*Bp* 14-1) was consistently more cytotoxic for, and better able to multiply within, macrophages than the parent (data not shown). In addition, *Bp* 14-1 was phagocytosed fivefold better, and the 8-h recovery of viable cells was more than twice that of the 1106a parent (Supplementary Figure 1). The progressive change from the parent of in vivo isolates in regard to macrophage virulence may be strain associated. For example, early isolates of *Bp* HBPUB10134a, a strain with more than a three-log greater IP virulence than 1106a [4], did not differ significantly from the parent in macrophage survival and cytotoxicity (data not shown).

The macrophage phenotypes of spleen isolates from mice with longer term infections were then characterized. *Bm* FMH isolates collected at days 26 and 45 after aerosol challenge differed only minimally in cytotoxicity and survival compared to the parent. However, day 60 isolates were distinctly reduced in survival and/or several cytotoxicity parameters, as illustrated in Table 4 and data not shown. The cytotoxicity induced by the parent strain was dose related in four of five experiments; a relatively low MOI produced significant bacterial survival and cell cytotoxicity in one experiment for unknown reasons.

**Table 4:** In vitro macrophage-associated phenotypes of isolates from *B*. *mallei*-infected animals

Strain^a^	Expt.	MOI	Bacterial survival (%)^b^	Cytotoxicity^c^		Cell morphology^d^	
				% cell loss (TB, DQ)	% dead (PI or TB stain)	Relative proportion MNGC	Major nuclear morphology
*Bm* FMH parent	9	7.7	0.90	>50	~50	≥10 %	<50/>50 % normal/cytotoxic
	11	2.9	5 × 10^−4^	<1	1–2	Rare, <1 %	>90/<10 % normal/cytotoxic
	12	4.3	~1 × 10^−4^	<1	<1	None	>90/<10 % normal/cytotoxic
	36	9.6	0.20	~40	~65	16.5	37/63 % normal/cytotoxic
	63	2.3	8.80	90	87	20.6	36/64 % normal/cytotoxic
Isolate 60-5	9	7.9	0.70	NC	**12**	**Rare**, **<1** **%**	**>50**/<50 % normal/cytotoxic
	11	2.3	5 × 10^−4^	NC	<1	**None**	NC
	12	3.9	**<1** × **10** ^**−5**^	NC	<1	NC	NC
	36	8.8	0.25	**~30**	~65	**7.10** **%**	47/53 % normal/cytotoxic
Isolate 60-3	63	2.2	**2.70**	**50**	**4**	16.8 %	38/62 % normal/cytotoxic

*NC* no change/same response as parent strain

^a^BALB/c mice were exposed by aerosol to parent strain *Bm* FMH in an LD_50_ experiment. Colony isolates were collected from spleens of surviving mice 60 days after exposure. Results are those of two isolates, 60-3 and 60-5, which had differed more from parent in other phenotypes (colony morphology and chemical susceptibility) compared to four other isolates. All were from two mice in the lowest aerosol dose group (32 CFU). Data are representative of seven experiments. Bolded values are those notably different from parent values

^b^The bacterial survival is expressed as the change in viable counts after 20–23 h compared to the input inoculum (% of input no. CFU)

^c^Cytotoxicity was determined after 20–23-h incubation and is shown as the % cells lost [by trypan blue and Diff-Quik™ (DQ) stains]. The % dead cells was determined by PI and trypan blue staining. The values do not include cells/MNGCs in an advanced state of necrosis with degraded, unstainable DNA

^d^The values describe approximate proportion of MNGCs among total cells/field and the major relative appearance of nuclei [typical large and intact vs. cytotoxic (necrotic/apoptotic or within an MNGC)]

**Fig. 3:**
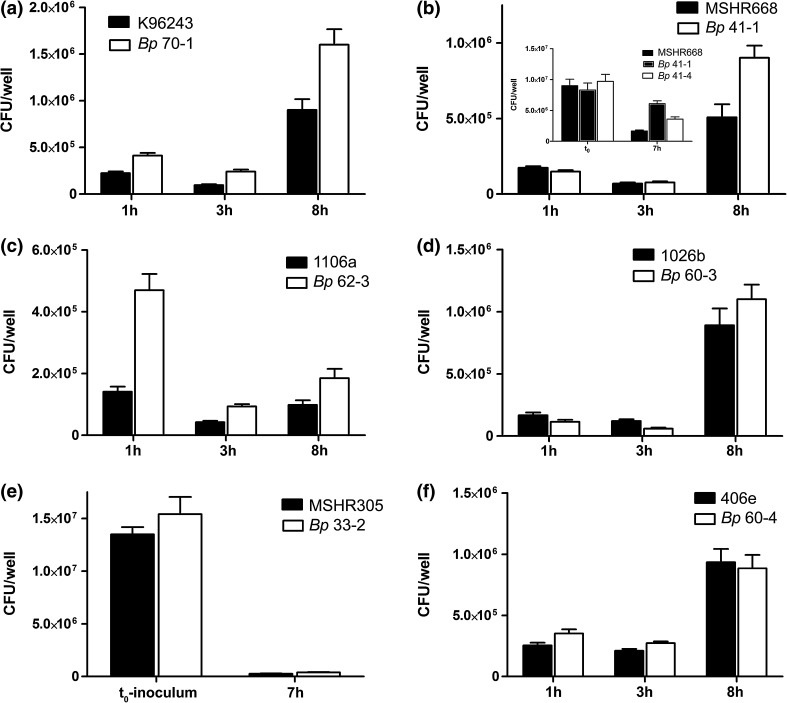
Comparison of macrophage survival of *Bp* parent strain and spleen isolate pairs. J774.A1 cells were incubated with the parent strain or isolate for 1 h. For **a**–**d**, **f**, the data shown are the mean viable counts (triplicate wells) recovered after 1-h uptake, after incubation of the infected cells in the presence of kanamycin for 2 h to remove unphagocytosed bacteria (3 h total), and after incubation for a total of 8 h. **a** *Bp* strain K96243 and a mouse spleen isolate collected 70 days after exposure (*Bp* 70-1) with MOIs of 13.3 and 11.0, respectively. The viable counts determined at the three times points were statistically different (1 and 3 h: *p* < 0.0001; 8 h: *p* = 0.0016). **b** *Bp* strain MSHR668 and mouse spleen isolates. The MOIs were 38.5 (MSHR668) and 32.3 (*Bp* 41-1). Each *set of bars* are the data for the challenge strain (MSHR668) and for a spleen isolate from a mouse surviving aerosol challenge and euthanized on day 41 (*Bp* 41-1); *p* = 0.004 for 8 h. *Inset* macrophages were infected with strain MSHR668 or isolates 41-1 or 41-4 at MOIs of 12.2, 11.2, and 13.1, respectively. The infected cells were incubated and treated as described above, and the viable counts obtained after a total of 7 h are shown. They are compared to the initial no. of CFU added to the wells (t_0_ inoculum); *p* < 0.001 for 7 h for 41-1. **c** *Bp* strain 1106a and a mouse spleen isolate collected 62 days after aerosol exposure (*Bp* 62-3), at MOIs of 12.3 and 14.1, respectively. The viable counts of *Bp* 62-3 determined at the three times points were significantly greater than those of 1106a (1 and 3 h: *p* < 0.0001; 8 h: *p* = 0.013). **D**. *Bp* strain 1026b and a spleen isolate from a mouse surviving aerosol challenge and euthanized on day 60 (*Bp* 60-3). The MOIs were 9.5 and 11.4, respectively. The viable counts of the parent strain were slightly greater than those of the isolate after 1-h incubation (*p* = 0.08, not significant) and after 3-h incubation (*p* = 0.008). Greater quantities of isolate than parent were recovered after 8 h although the absolute differences were not statistically significant. **e** Macrophages were infected with strain MSHR305 or a spleen isolate collected 32 days after exposure (*Bp* 33-2), at MOIs of 15.4 and 17.5, respectively. The infected cells were incubated for 1 h and treated with kanamycin for 2 h, and the viable counts obtained after a total of 7 h are shown. The quantities of bacteria of the two strains recovered from the cells at 7 h were not significantly different (1.7 and 2.5 %, respectively). **f** *Bp* strain 406e and mouse spleen isolate *Bp* 60-4 at MOIs of 17.1 and 15.5, respectively. The viable counts obtained at all three time points for the parent and isolate were not significantly different (*p* > 0.05). The observed differences for these experiments were confirmed using comparisons of the normalized viable CFU counts

In contrast to the results with *Bm*, the survival and cytotoxicity of the isolates in comparison to the *Bp* parent appeared to be strain dependent. Of the strains of *Bp* from which isolates from long-term infections could be recovered, a trend toward enhanced macrophage virulence was observed consistently for strains 1106a and MSHR668 and typically, but less consistently, for K96243. The responses of the 1026b isolates were variable, and there were usually no differences observed for isolates of parent strain 406e (Fig. 3). Figure 3a shows the results of a spleen isolate recovered on day 70 after aerosol exposure with strain K96243. Isolate *Bp* 70-1 was phagocytosed to a greater extent compared to K96243 for all three time points. Greater cell death and detachment was also associated with *Bp* 70-1 at 8-h (Table 5). The greater cell loss associated with *Bp* 70-1 at 8-h suggests that the 8 h viable counts might have underestimated the strain’s replication. To account for differences in strain MOIs, the counts were normalized to the inoculum (no. CFU added at t_0_) and expressed as percentages. These normalized values, presented in Supplementary Table 7, confirmed the differences shown here. Isolates recovered 49 days post-exposure also exhibited better survival than the parent K96243 strain (data not shown).

**Table 5:** Phenotypes of macrophages infected with *B*. *pseudomallei* parent and spleen isolates—cytotoxicity comparisons

Strain	Panel^a^	MOI	% cell loss^b^	% cells dead (TB)^b^	No. MNGC or necrotic cells (% of total)^c^	No. MNGC nuclei (% of total)^c^
K96243	a	13.3	40–50	17	4.4	43.6
*Bp* 70-1		11.0	60–65	32.4	5.8	58.8
MSHR668	b inset					
expt. #1		12.2	35–40	4.5	7.6	32.0
*Bp* 41-1		11.2	60–70	20.0	13.0	54.0
*Bp* 41-4		13.1	35–40	12.5	14.0	58.0
MSHR668	b					
expt. #2		38.5	20	nd	20.7	67.8
*Bp* 41-1		32.3	18	nd	18.0	73.2
1106a	c	12.3	40	27.9	21.9	70.8
*Bp* 62-3		14.1	60–65	73.2	41.8	85.4
1026b	d	9.5	25–30	24	8.8	35.3
*Bp* 60-3		11.4	25–30	25	9.6	47.4
MSHR305	e	15.4	30	2.5	3.5	10.0
*Bp* 33-2		17.5	35	2.5	3.5	10.0
406e	f	17.1	40	10.3	5.6	21.8
*Bp* 60-4		15.5	45	22.3	5.0	25.8

*nd* not done

^a^Figure 3, panels a–f

^b^Cytotoxicity is shown as the % cells lost from the macrophage layer by trypan blue (TB) staining and the % dead cells among those remaining by TB and/or PI staining. However, necrotic cells and advanced-stage MNGCs with degraded nuclei are unstained by TB, and the values underestimate the actual proportion of dead cells

^c^The values describe the approximate relative proportion of MNGCs (%) among the total cells in six fields, two from each of three replicate coverslips (×600); and the major nuclear phenotype, considering all the nucli in the fields (typical large nuclei vs. nuclei present in MNGC with intact stained nuclei or in necrotic fused cell masses with lightly stained nuclei), expressed as percentages. Counts were done using Diff-Quik-stained cells. The more extensive cell death and detachment induced by some strains may minimize the extent of these values

**Fig. 4:**
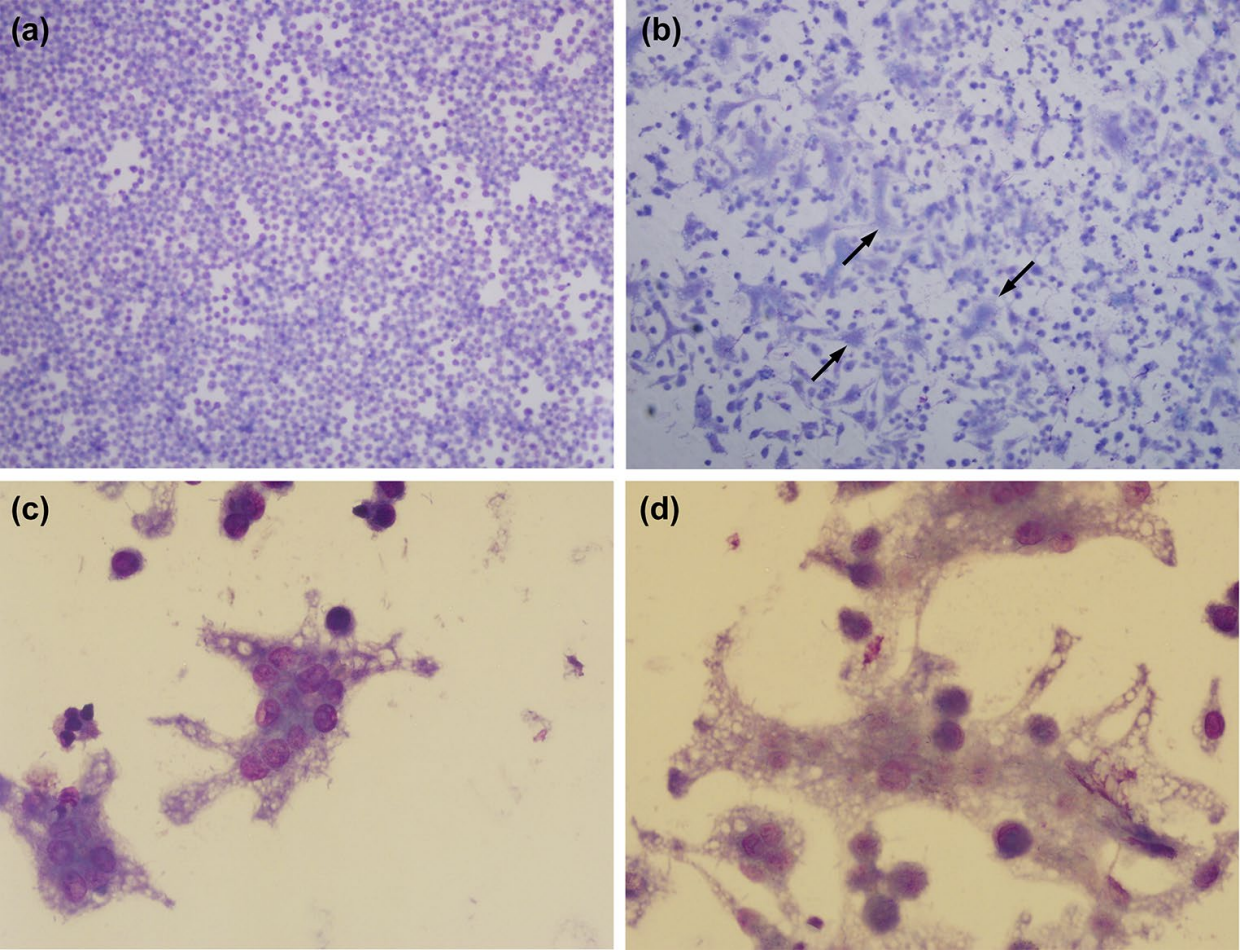
Changes in the morphology and adherence of macrophages infected with *Bp*. Cells were infected with spleen isolate *Bp* 41-1 of strain MSHR668 and stained with Diff-Quik™ after 7-h incubation. The cultures are shown under low power (×100, **a**, **b)** and higher power (×600, **c**, **d**). **a** Uninfected cells of normal semi-confluent J774.A1 cells. **b** Cells infected with isolate *Bp* 41-1 showing detachment/loss of cells and presence of numerous multi-nucleated giant cells (MNGCs, indicated by *arrows*) and clusters of fused necrotic cells. **c**, **d** Cells infected with isolate *Bp* 41-1 and illustrating the appearance of MNGCs with numerous intact nuclei (**c**) and MNGCs in a more advanced state of decay with more necrotic, poorly stained nuclei (**d**)

In experiments with strain MSHR668 and two day 41 isolates, the isolates again replicated better than the challenge strain (Fig. 3b; Supplementary Table 7); 7–8 h counts of the isolates were significantly greater than those of the parent. The isolates were more cytotoxic at lower MOIs, as shown by the greater proportion of TB-stained dead cells and the greater number and larger size of MNGCs (Table 5). The cytotoxic effects on macrophages often associated with *Bp* infection are illustrated by MSHR668 isolate *Bp* 41-1-infected cells in Fig. 4. The detachment of J774.A1 cells and the formation and appearance of MNGCs are shown in panels A–B and C–D, respectively. Isolates recovered 60 days after IP challenge exhibited similar macrophage phenotypes (data not shown). The results with strain 1106a and an isolate collected 62 days after challenge again exemplified the increase in isolate macrophage infectivity and cytotoxicity, as shown in Fig. 3c. Isolate *Bp* 62-3 was phagocytosed to a greater extent. Both strains doubled during the 5-h incubation after removal of antibiotic, but the greater loss of macrophages during this time in the *Bp* 62-3-infected culture likely resulted in an underestimation of the 8 h viable counts. The isolate was more cytotoxic for the macrophages, inducing more cell death and detachment from the wells (Table 5).

As shown in Fig. 3d and Table 5, a day 60 isolate from strain 1026b was variably more active in macrophages than the parent strain, albeit marginally. Although *Bp* 60-3 was phagocytosed to a slightly lesser extent than 1026b (1-h and 3-h viable counts), the isolate replicated more than twice as fast as the 1026b parent post-phagocytosis, as determined by the greater normalized 8-h counts (742 vs. 1839 % of the inoculum for parent and isolate, respectively; Supplementary Table 7). Isolate *Bp* 60-3 was also slightly more cytotoxic, as shown by the MNGC parameters (Table 5 and data not shown). The macrophage phenotypes of strain MSHR305 and an isolate collected 33 days after aerosol challenge (*Bp* 33-2) suggest the isolate was similar to the parent in its capacity to replicate in the macrophages and induce a cytotoxic response (Fig. 3e; Table 5). Additional day 33 isolates exhibited variable macrophage phenotypes (data not shown), perhaps suggesting that persistent infection had not been established. Finally, the spleen isolates evaluated from mice challenged with *Bp* strain 406e did not induce macrophage phenotypes that were consistently different from the challenge strain, as determined with spleen isolates from days 34 and 60 after infection (Fig. 3f; Supplementary Table 7; Table 5; and data not shown.

#### LPS phenotyping

**Fig. 5:**
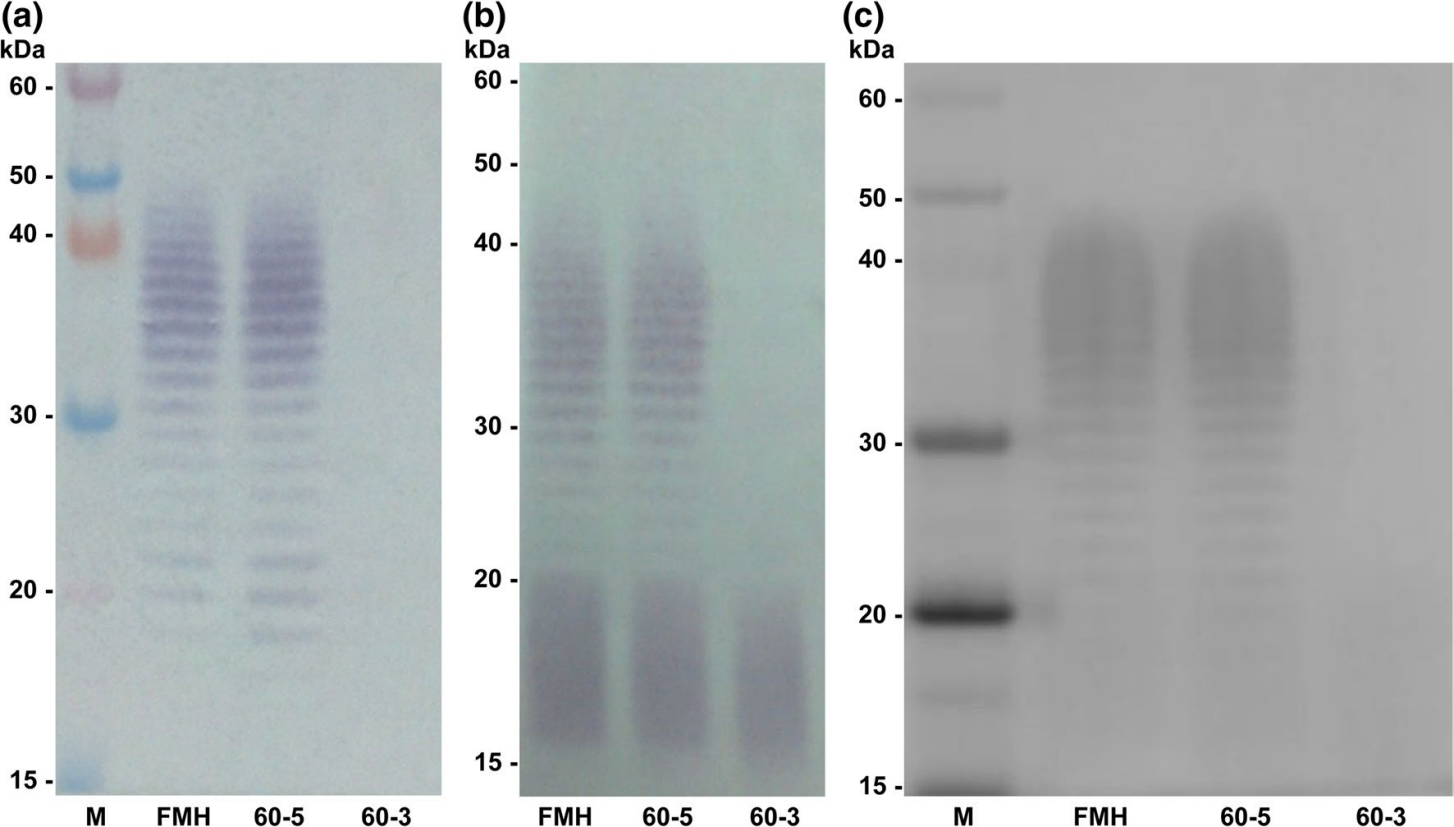
LPS phenotyping comparing the *Bm* FMH parent strain with two 60-day mouse spleen isolates. Western blots using monoclonal antibody 8G3-1B11 (**a**) and polyclonal antibody ABE 335 (**b**) show the loss of O polysaccharide in isolate 60-3. This LPS change was confirmed by silver staining (**c**)

LPS phenotyping was explored as an additional tool for distinguishing different strains of *Bp* and *Bm* as well as their in vivo isolates. Distinct strain-specific differences in the LPS banding profiles of numerous other strains of each species were distinguished previously (Welkos et al. 2015). In this study, LPS electrophoretic banding profiles of 20 *Bp* and 13 *Bm* isolates were compared to those of their respective parent strains using species-specific monoclonal antibodies for LPS (11G3-1 for *Bp* and 8G3-1B11 for *Bm*). One spleen isolate of *Bm* FMH obtained on day 60 post-infection displayed a change in LPS phenotype, from smooth to rough, during the course of infection (Fig. 5). Figure 5a shows a western blot with parent strain FMH, a day 60 isolate that did not change its LPS phenotype (60-5), and the 60-3 isolate that appeared to change from smooth to rough. The transition from a smooth to rough LPS phenotype results from the loss of O polysaccharide (OPS). This result was further supported by a western blot using polyclonal antibody ABE 335 where OPS was absent and lipid A was visible, confirming the presence of LPS (Fig. 5b). To rule out the possibility that the OPS simply changed its structure, thus preventing antibody recognition, silver staining was performed (Fig. 5c). No OPS could be detected for 60-3 by silver straining, thereby confirming the rough phenotype.

#### Comparison of mouse virulence of *Burkholderia* strains and their spleen isolates

**Fig. 6:**
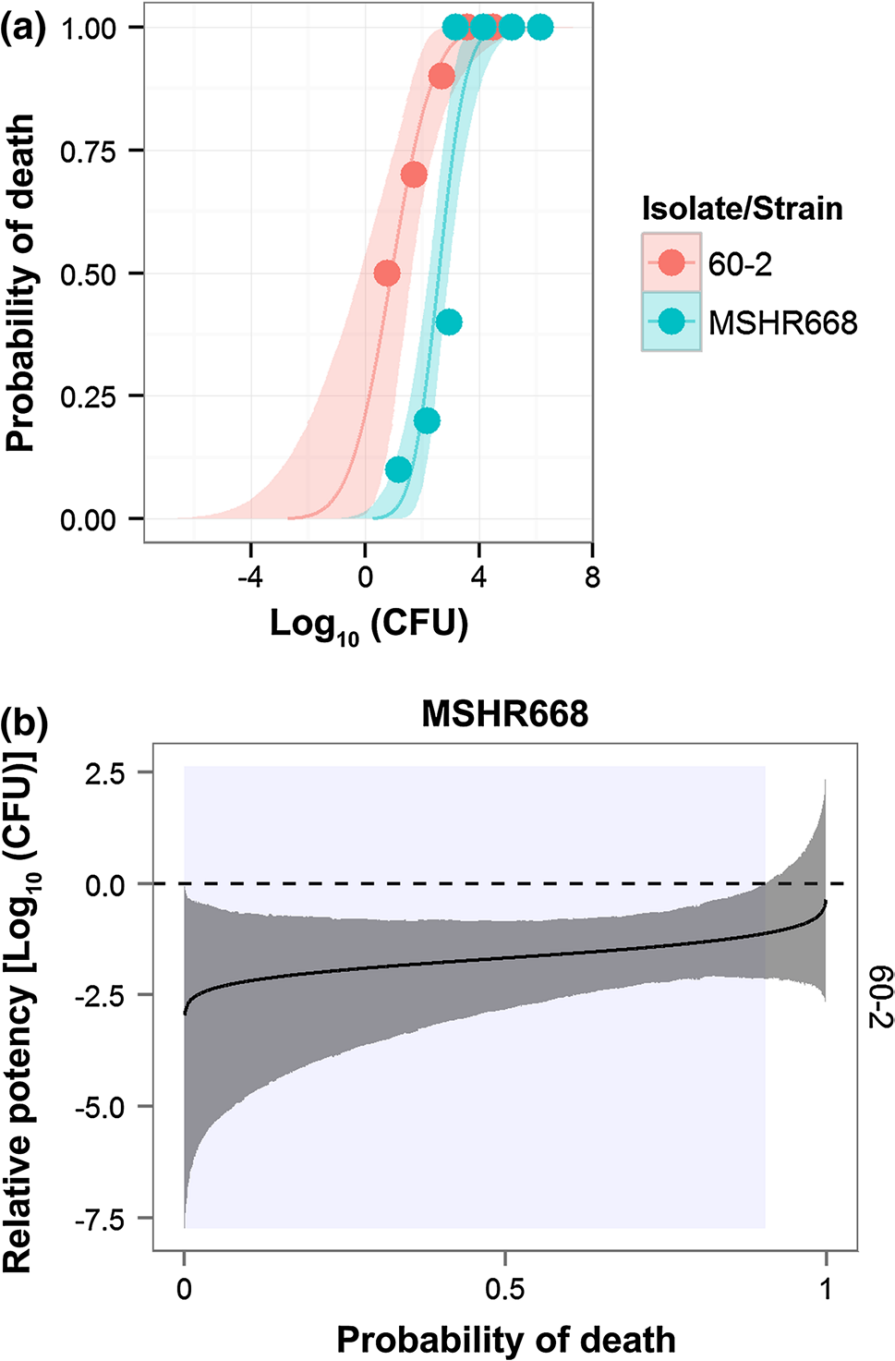
Comparison of relative virulence for mice of *Bp* strain MSHR668 and spleen isolate *Bp* 60-2 collected on day 60 after infection. **a** Mice were challenged by the IP route with different doses of the bacteria, and the daily morbidity/mortality data through day 60 were used to determine the strain LD_50_ values. The isolate LD_50_ was significantly less than that of the parent (Table 6). **b** Statistical pairwise comparisons of relative potency of MSHR668 and its isolate at each lethality level along the dose–survival curve

An initial study to compare the virulence and morbidity of strains of *Bp* and *Bm* with their in vivo isolates was conducted in an established *Burkholderia* mouse model (Welkos et al. 2015). In the current study, BALB/c mice were challenged by the IP route with dilutions of a strain of *Bp*/*Bm* or with a selected spleen isolate. The LD_50_ values and relative virulence potencies of parent versus isolate were evaluated statistically using the survival data accrued by days 21 and 60 (Table 6). Isolates collected from midterm survivors did not always show statistically significant differences from their respective parent strains. However, the day 60 LD_50_ of K96243 isolate *Bp* 49-4 was tenfold less, suggesting an enhanced virulence of the isolate compared to the parent strain. As for the long-term survivors, *Bp* 60-2 was significantly more virulent than its parent strain MSHR668, i.e., its day 21 LD_50_ was 46-fold less than that of the parent strain (Table 6; Fig. 6a). The greater virulence of the isolate was also demonstrated by its greater potency at all lethal doses up to 90 % mortality (probability ≤0.9). In Fig. 6b, the lightly shaded area indicates the range of lethal doses of *Bp* 60-2 that were associated with significantly greater virulence compared to MSHR668. Larger doses of MSHR668 were required to produce the same lethality achieved with lower doses of isolate. Finally, even though the day 21 LD_50_ values of the parent *Bm* FMH strain and isolate *Bm* 60-5 were similar, the day 60 LD_50_ for isolate *Bm* 60-5, albeit not statistically significant, was almost fourfold less than that of parent strain (Table 6).

**Table 6:** Statistical comparison of relative virulence for mice of *Burkholderia* strains and their in vivo isolates

Strain	Day 21^a^			Day 60^a^		
	LD_50_	95 % credible interval		LD_50_	95 % credible interval	
		Lower	Upper		Lower	Upper
*Bp* MSHR668	3.9 × 10^2^	1.7 × 10^2^	8.6 × 10^2^	0.3	0	1.6 × 10^1^
*Bp* 41-1	4.7 × 10^2^	1.2 × 10^2^	2.2 × 10^3^	5.1	0.1	3.8 × 10^1^
*Bp* 60-2	**8.4** ^b^	0.7	4.3 × 10^1^	3.4	0.5	1.2 × 10^1^
*Bp* K96243	2.3 × 10^4^	1.2 × 10^4^	4.7 × 10^4^	1.9 × 10^3^	4.7 × 10^2^	6.1 × 10^3^
*Bp* 49-4	4.3 × 10^4^	2.3 × 10^4^	7.7 × 10^4^	**1.9** **×** **10** ^**2**c^	0.4	2.2 × 10^3^
*Bp* 1106a	3.3 × 10^4^	1.7 × 10^4^	6.4 × 10^4^	1.2 × 10^4^	3.3 × 10^3^	4.0 × 10^4^
*Bp* 62-3	4.7 × 10^4^	1.9 × 10^4^	1.2 × 10^5^	1.2 × 10^4^	7.0 × 10^3^	2.0 × 10^4^
*Bm* FMH	3.3 × 10^7^	1.4 × 10^7^	7.6 × 10^7^	1.1 × 10^7^	2.1 × 10^6^	7.2 × 10^7^
*Bm* 60-5	2.1 × 10^7^	1.2 × 10^7^	3.7 × 10^7^	**2.9** **×** **10** ^**6**d^	7.7 × 10^5^	1.2 × 10^7^

^a^BALB/c mice were challenged by the IP route with a strain of *Bp* or *Bm*, or a spleen isolate of the strain, and the mice were monitored for morbidity and mortality for 60 days. The 21- and 60-day survival data were analyzed for each strain, and the data were evaluated statistically to compare parent strain and spleen derivative relative virulence potencies and LD_50_ values (no. CFU) by using Bayesian probit analysis, as described in the methods. The bolded LD_50_ values are those which were significantly different from the parent strain LD_50_ value

^b^The day 21 LD_50_ value of *Bp* 60-2 was less than that of parent strain MSHR668 (probability ≥95 %) and the isolate exhibited greater potency at all lethal doses up to 90 % (probability of mortality ≤0.9)

^c^The day 21 LD_50_ values of K96243 and its isolate *Bp* 49-4 were not significantly different. The day 60 LD_50_ of the isolate was about tenfold less than that of the parent, although not significantly different

^d^Although the day 60 LD_50_ for isolate *Bm* 60-5 was almost fourfold less than that of the parent strain, the values were not significantly different

## Discussion

A number of in vitro assays were used to identify potential phenotypic markers for chronic *Bp* and *Bm* infection. Although a relatively small number of strains were fully characterized in vitro and over an extended period in vivo, the strains described represented a diversity of *Bp* from different patient sources and geographical locations to include the two foci that have been of predominant research concern: Thailand and Northern Australia. Characterization of bacteria isolated during the later stages of a long-term infection with *Bp* or *Bm* revealed frequent differences in all of the characterized phenotypes of the isolates compared to the original parent strains. The results of antimicrobial sensitivity assays commonly showed an increase in resistance in both *Bm* and *Bp* isolates. Whereas there was evidence for a positive association between development of increased resistance to particular antibiotics, chemicals, and AMPs in *Bp* isolates recovered at early compared to late infection times, there was no such trend clearly established with *Bm* isolates. In macrophage assays, infectivity/cytotoxicities of *Bp* isolates from mice with extended infections were greater than that of the parent. In contrast, *Bm* isolates were reduced in macrophage replication and cytotoxicity. In LPS phenotypic analysis, a potential loss of OPS expression in *Bm* was discovered during the course of infection. Finally, preliminary studies in a mouse model suggested that enhanced pathogenicity was associated with phenotypic variation of isolates from persistent infections.

Many host cells which provide a niche for persistent *Burkholderia* infection also possess activities and antibacterial products (e.g., AMPs and reactive oxygen and nitrogen species) important in innate immunity. Thus, intracellular pathogens would need to overcome such defenses. In our antimicrobial sensitivity assays, differences in the sensitivities to certain antibiotics, chemicals, and AMPs between isolates and parent *Bp* and *Bm* strains were often observed. Antimicrobial peptides are small cationic molecules which are part of the innate host defenses of many organisms. These AMPs are not specific for a particular target and their bactericidal activity usually involves membrane disruption (Andreu and Rivas 1998; Fox et al. 2012; Higashijima et al. 1988; Kreil 1973; Madhongsa et al. 2013; Skerlavaj et al. 1996; Steiner et al. 1981; Vila-Farres et al. 2012; Zasloff 2002, 1987). They are potential therapeutic agents for antibiotic-resistant organisms such as *Bp* and *Bm* (Kanthawong et al. 2009; Kreil 1973; Madhongsa et al. 2013). However, *Burkholderia* spp. have been reported to be relatively resistant to AMPs, although the sensitivity of *Bp* appears to be strain-specific (Fox et al. 2012; Kanthawong et al. 2009; Tandhavanant et al. 2010). For instance, notable sensitivity of *Bp* to the human cathelicidin peptide LL-37, or to hybrid peptides containing this AMP, has been observed (Fox et al. 2012; Kanthawong et al. 2009; Tandhavanant et al. 2010). Although the *Bp* and *Bm* strains used in this study were overall relatively resistant to AMPs, they were often more sensitive to some AMPs than were their spleen isolates (i.e., For mastoparan 7, BMAP-18, and magainin). Therefore, altered sensitivities of isolates to certain AMPs, such as mastoparan 7 and BMAP-18, could potentially be used as candidate markers for screening isolates from persistent *Burkholderia* infections. Nonetheless, and as expected, increased isolate resistance to specific AMPs was strain dependent, as demonstrated for 1026b and K96243.

Intracellular *Bp* has also been shown to evade or suppress production of reactive oxygen and nitrogen species, as well as other harsh chemicals (Chantratita et al. 2007; Dowling et al. 2010; Loprasert et al. 2003; Mulye et al. 2014; Tandhavanant et al. 2010; Utaisincharoen et al. 2001, 2004). In our study, *Bp* spleen isolates from three strains harbored sensitivities to sodium nitrite that differed from the parent. But whereas isolates of one strain were more resistant than the parent, those from two strains exhibited increased sensitivity. However, for most chemicals, the in vivo isolates displayed phenotypes of enhanced resistance, as exemplified by the responses to elevated NaCl concentrations, potassium tellurite, and variably to the surfactant Niaproof 4. Detergents such as Niaproof 4 often have opsonophagocytic activity and may play a role in innate immunity (Wright 1997, 2003). A direct association between time post-challenge and the number or extent of sensitivity differences exhibited by isolates was not always apparent but was suggested for not only several AMPs, but also the antibiotic nalidixic acid and potentially for Niaproof 4 and potassium tellurite, as described above. The isolate response data *en toto* suggested that extended maintenance of *Bp* in vivo may be facilitated by development of an increased resistance to antibacterial environments such as the macrophage phagolysosome.

As illustrated by our findings, the identification of infection-associated in vitro phenotypes is influenced by the phenotypic variability of the *Burkholderia*, and especially *Bp*, which is often associated with reversible epigenetic alterations (Chantratita et al. 2007, 2012; Velapatino et al. 2012; Vipond et al. 2013). The ability of *Bp* to produce different colony morphotypes is well known (Nicholls and Cantab 1930; Rogul and Carr 1972; Stanton et al. 1924) and potentially reflects adaptive changes that enhance fitness in a particular environment. Chantratita et al. (2007) observed that different *Bp* colony morphologies could be recovered from individual sites in patients with melioidosis. They identified and characterized seven colony morphologies. Type I could switch to the other six types in apparent response to environmental stresses such as iron limitation (Chantratita et al. 2007). Morphotypes I, II, and III were associated with differential ability to survive and persist in cell culture and mice and with resistance to peroxide and AMPs (Chantratita et al. 2007; Tandhavanant et al. 2010). Morphotypes associated with adaptive fitness differed significantly in the expression profiles of up- and down-regulated proteins (Chantratita et al. 2012). However, the finding that specific colony morphotypes are predictive of enhanced resistance to certain stresses may be strain related. Analyses of additional *Bp* strains revealed heterogeneity in responses between and within strains (Chantratita et al. 2007; Tandhavanant et al. 2010), and further analyses may be needed to confirm associations between in vitro phenotype differences and in vivo survival and persistence. More recently, Wikraiphat et al. (2015) described mucoid (M) and nonmucoid (NM) variants from the same sample which exhibited antigenically different OPS. The association between colony type switching and conformational differences in OPS was determined in analysis of several M/NM pairs from clinical samples under different in vitro growth conditions. Although the mechanism regulating colony/OPS variation and differential growth fitness is not entirely clear, the association between colony type and OPS modification might be the basis of the colony variation described previously (Nicholls and Cantab 1930; Srinivasan et al. 2001). As reported by Austin et al. (2015), *Bp* strain K96243 exhibited three colony variants which differed not only in colony morphology but also in other in vitro phenotypes. The isogenic colony types (one white and two yellow forms) were relatively stable but reversible, and they grew differentially in vitro under low oxygen or acidic environments. Only one type, albeit highly attenuated, could persist in the harsh conditions of the murine stomach and colonize the mucosa. A transcriptional regulator controlling colony variation was identified, though it is unknown whether this gene or a global master regulator controls this as well as other examples of reversible phenotypic switching in *Bp*. Lastly, Velapatino et al. (2012) analyzed proteins from *Bp* isolates recovered from a patient during the acute and relapse stages of infection. Colony Morphotype I (Chantratita et al. 2007; Wikraiphat et al. 2015) was recovered from the primary infection and an additional morphotype (III) was recovered during the relapse phase. Many proteins were differentially regulated for the relapse isolate compared to acute stage isolates, e.g., enhanced production of factors which detoxify the damaging RNI nitric oxide and proteins required for anaerobic growth. Prospective studies with *Bp* strains harboring mutations in relevant differentially expressed genes are needed to link expression with in vivo persistence. Strains of *Burkholderia* species other than *Bp* are also known to exhibit phenotypic variability. Romero et al. (2006) showed that in vitro passaging of *Bm* strains originally obtained from animal or human sources produces a diverse population of clones with alterations in mRNA levels of many genes. The changes in phenotypic expression were associated with insertions or deletions in DNA repeat regions which occurred rapidly. Such events may contribute to the instability of the *Bm* genome and enhance bacterial fitness. They might also partially explain the variability observed in this and other studies between and within *Bp* and *Bm* strains in colony morphology and in response to antimicrobial compounds (Austin et al. 2015; Chantratita et al. 2007; Tandhavanant et al. 2010; Velapatino et al. 2012; Wikraiphat et al. 2015). Members of the *Burkholderia cepacia* complex (BCC) exhibit colony morphology switching associated with their ability to colonize the lungs and cause severe infection in patients with cystic fibrosis (Bernier et al. 2008; Vial et al. 2010). For instance, phase switching by *B*. *ambifaria* resulted in alterations in colony morphology, biofilm formation, plant root colonization, and virulence. The data suggested that *B*. *ambifaria* adapts to the very different environments of patient lungs and the rhizosphere by reversible phase variation.

Phagocytic components of the cellular immune system are known to have a dual role in infection. They can serve both as critical components of host immune protection against *Burkholderia* infection and as facilitators of bacterial persistence in vivo (Galyov et al. 2010; Gregory and Waag 2008; Mulye et al. 2014; Valvano et al. 2005). For instance, depleting mice of macrophages has been shown to increase their susceptibility to melioidosis (Breitbach et al. 2006), and inducible nitrous acid synthase was essential for clearance of *Bm* from macrophages (Brett et al. 2008). These results suggest a role for macrophages in host protection. However, *Bp* and *Bm* produce virulence factors (secreted proteins of the T3SS and T6SS-1, BimA, and others) which allow the bacteria to escape the phagolysosome, multiply in the cytoplasm, and spread by direct cell-to-cell passage (Brett et al. 1994; Chuaygud et al. 2008; Dowling et al. 2010; Galyov et al. 2010; Gregory and Waag 2008; Kespichayawattana et al. 2000; Stevens et al. 2005a, b; Utaisincharoen et al. 2004; Valvano et al. 2005; Whitlock et al. 2008, 2009). Thus, the expression of numerous virulence factors which promote bacterial survival and inhibit cellular antibacterial mechanisms may contribute to the persistence of these agents in a relatively sheltered intracellular host niche (Butt et al. 2014).

Several studies have highlighted genetic and phenotypic characteristics potentially associated with persistence of *Bp* and *Bm* (Hayden et al. 2012; Price et al. 2013; Velapatino et al. 2012). Hayden et al. compared genome sequences of *Bp* strain pairs from patients consisting of the primary infecting strain and an isolate from relapsed infection occurring long after a successful response to antibiotic treatment (Hayden et al. 2012). They observed numerous genetic changes (indels, SNPs, and structural variations) for all the primary-relapse pairs. The only consistent alteration was in a homolog of the TetR family of global transcriptional regulators. At least one of the relapse isolates exhibited much higher MICs to several antibiotics relative to the primary strain. Their studies supported the hypothesis that this homolog and similar genes play a role in *Bp* adaptive survival in vivo. In studies with *Bm*, Romero et al. reported extensive genomic variability and rapid alterations in RNA expression in isolates obtained after passage in animal and human subjects (Romero et al. 2006). Price et al. also found significant genomic changes in *Bp* strains isolated almost 12 years apart from a chronically infected human survivor (Price et al. 2013). These changes appeared to mark an adaptive evolution that occurred over time and included inactivation of major virulence factor and regulatory genes required for environmental survival. This apparent reductive evolution appeared to mimic the process thought to have led to the genomic losses and conversion of *Bm* from a free-living microbe such as *Bp* into an obligate host pathogen.

In this study, although isolates of *Bm* FMH obtained early after infection displayed variable macrophage cytotoxicity and survival, most collected on day 60 were clearly reduced in both toxicity and intracellular growth. The basis for the attenuated phenotype is not known, but it conceivably reflects a shift in the infection toward a more chronic or subclinical intracellular form less prone to stimulate host immune responses. In contrast, of the five *Bp* strains for which colonies from mid- to long-term infections were available, isolates from three (MSHR668, 1106a, and less consistently, K96243) were more virulent for macrophages than the parent strain in their ability to multiply within or produce cytotoxic responses. However, the other *Bp* strains were more variable so the significance of the relationship between macrophage phenotypes and mouse virulence requires further study. It must be noted that macrophage cultures cannot replace or fully model pathogenesis, and animal models are needed for preliminary confirmation of significant findings obtained from cell culture. Furthermore, results using macrophage models may differ depending on the source of macrophage used and other factors. Whereas the results shown here and those of various other investigators employed murine-derived macrophage-like J774.A1 cells (Chantratita et al. 2007, 2012; Haussler et al. 2003; Kespichayawattana et al. 2000; Stevens et al. 2003; Tandhavanant et al. 2010; Wand et al. 2011; Welkos et al. 2015), others used cell lines such as murine RAW264.7 cells or human monocyte-like U937 cells (Arjcharoen et al. 2007; Brett et al. 2008; Burtnick et al. 2010, 2011; Chantratita et al. 2012; Pegoraro et al. 2014; Stevens et al. 2002; Tandhavanant et al. 2010; Utaisincharoen et al. 2000, 2004). Overall, we observed relatively reduced survival of *Bm* in J774.A1 macrophages compared to that of *Bp*, as reported previously (Brett et al. 2008). However, *Bm* generally appeared to be capable of greater J774.A1 macrophage survival and extensive cytotoxic activity at higher MOIs, yet these attributes were negatively impacted at higher MOIs in studies using a RAW264.7 model (Brett et al. 2008). The source of the macrophages and differences in model parameters likely explain some of the differences in results. Macrophage models are important tools in pathogenesis research with the caveat that they likely provide a model which is restricted to specific defined aspects of infection.

LPS differences can also be used as phenotypic markers, and previous studies suggest that *Burkholderia* LPS changes could be associated with chronic infection (Evans et al. 1999; Price et al. 2013). In this study, we provide evidence that *Bm* has the ability to lose OPS during the course of infection. This is intriguing because OPS modification and loss has been established as a hallmark of chronic *Pseudomonas aeruginosa* infection (Smith et al. 2006). In addition, rough *Burkholderia cepacia* strains lacking OPS have been associated with chronic infection in the lungs of cystic fibrosis patients (Evans et al. 1999). There are many possible reasons why losing OPS could benefit *Burkholderia* during infection. *P*. *aeruginosa* and *Salmonella typhimurium* mutants lacking OPS have been shown to increase outer membrane vesicle (OMV) formation (Salkinoja-Salonen and Nurmiaho 1978; Smit et al. 1975). OMVs are important virulence factor delivery mechanisms used by a wide variety of bacterial pathogens and can help the bacteria disseminate throughout the body (Kuehn and Kesty 2005). OMVs are also secreted by *S*. *typhimurium* when grown intracellularly (Garcia-del Portillo et al. 1997). In addition, once inside the macrophage, the bacteria may no longer have a need for OPS. *Bp* and *Bm* OPS has been shown to play a role in serum resistance (Brett et al. 2007; DeShazer et al. 1998). However, a mutation that disrupts OPS synthesis may not have a negative effect on bacterial survival once inside the macrophage because the bacteria are protected from serum. The loss of OPS could also play a role in evading the host immune response during relapse of an infection. OPS is highly immunogenic, and antibodies are produced by the host to recognize OPS from specific bacterial species, including *Bp* and *Bm* (Ho et al. 1997; Trevino et al. 2006). The loss of OPS could be highly advantageous during relapse of infection when the bacteria exit the macrophage, as the immune system would have a delayed response in recognizing the bacteria. Due to these possible implications, it is clear that the potential role of OPS in *Burkholderia* chronic infection warrants further investigation. Future studies include sequencing the OPS biosynthetic gene cluster of *Bm* FMH 60-3 to identify the genetic basis for the loss of OPS. In addition, the LPS of isolates from more well-established chronic *Bp* and *Bm* mouse models will be examined to look for additional instances of LPS changes during the course of infection. Rough isolates/strains will also be passaged through mice to determine the effect of OPS loss on virulence.

In initial attempts to detect a relationship between altered phenotypes and pathogenicity, the mouse virulence of *Bp*/*Bm* strains and their respective isolates was compared. The challenge data for three strains and their in vivo isolates suggested that bacteria isolated from persistently infected animals can differ significantly from the original strain in virulence, as indicated by LD_50_ and lethal dose potency comparisons. This was clearly demonstrated with one isolate (MSHR668 isolate 60-2) and potentially for the isolates of *Bp* strain K96243 and *Bm* strain FMH. Taken together, the data suggest that some isolates from mice persistently infected with certain *Burkholderia* strains might be enhanced in virulence compared to the parent and that this enhanced pathogenicity was associated with phenotypic variation. It was interesting that isolates of two of the three *Bp* strains (MSHR668 and K96243), with a tendency for enhanced survival and cytotoxicity for macrophages, were also more virulent for mice. The inverse was observed for the day 60 *Bm* isolate 60-5 which was more virulent for mice but, as shown also for *Bm* isolate 60-3, proliferated less and/or was less cytotoxic for macrophages. Perhaps the different results of *Bp* and *Bm* in vivo isolates in macrophages are associated with the lesser ability of *Bm* to destroy the host cell (and lose an in vivo niche) compared to *Bp* in the J774.A1 macrophage model. Further investigations are obviously needed. However, it may be possible to take advantage of several of the measurable aspects of the bacterial-macrophage interaction to show that a significant change (increase/reduction) in a parameter of infection, such as a change in the proportion of MNGCs, represents a potential marker to signal a conversion of a relatively acute to more persistent stage in the pathogenesis of infection.

It is not unexpected that the virulence of the in vivo isolates often appeared to be enhanced compared to that of the parent strain. The laboratory culture of pathogenic bacterial strains has been shown to result in the accumulation of genetic changes and thus genetically mixed cultures which can impact virulence. Genomic plasticity and instability has been extensively characterized not only for *Bp* and *Bm* but also for *Yersinia pestis* (Buchrieser et al. 1998; Galyov et al. 2010; Perry and Fetherston 1997; Romero et al. 2006; Sahl et al. 2015; Tumapa et al. 2008). The passage of nonuniform cultures of some pathogens such as *Y*. *pestis* and *Legionella pneumophila* in vivo selects for the most fit clones and has been used to increase genetic uniformity and restore virulence (Bozue et al. 2011; Cirillo et al. 1999; Welkos et al. 1997). This has also been shown or suggested more recently for *Bp* and *Bm* (Chen et al. 2014; Inglis et al. 2000; Waag and DeShazer 2004). However, the *Burkholderia* appear to exploit their capacity for genomic variability by generating diverse populations which accumulate in vivo in response to differing host environments (Price et al. 2010, 2013; Romero et al. 2006). Thus, the impact of in vivo passage on the *Burkholderia* could be complex and strain or host dependent.

Overall, this study has identified potentially useful and often specific phenotypic markers of long-term *Burkholderia* infection in several different phenotypic classes (morphotype, antimicrobial sensitivities, macrophage infection, LPS, and murine virulence). Additional studies are needed to confirm the consistency and range of these phenotypes in chronic infection models and examine the potential role for them in establishment of a chronic infection. However, in vivo residence was clearly associated with changes in resistance and responses to many potential host antibacterial defenses. The wide diversity of the antibacterial chemicals and other stressors impacted by these altered *Bp* responses suggests that the host environmental factors and/or bacterial switch mechanisms driving a conversion to resistance are multifactorial.

## Electronic supplementary material

Below is the link to the electronic supplementary material.

Supplementary material 1 (DOCX 17 kb)

Supplementary material 2 (DOCX 18 kb)

Supplementary material 3 (DOCX 23 kb)

Supplementary material 4 (DOCX 23 kb)

Supplementary material 5 (DOCX 26 kb)

Supplementary material 6 (DOCX 17 kb)

Supplementary material 7 (DOCX 19 kb)

Supplementary Figure 1 Comparison of macrophage survival of *Bp* strain 1106a and mouse spleen isolate *Bp* 14-1. J774.A1 cells were inoculated with MOIs of 16.6 and 11.0, respectively, and incubated for 1 h. The data shown are the mean viable counts (triplicate wells) recovered after the 1-h uptake, incubation of the infected cells in the presence of kanamycin for 2 h (3 h), and after incubation for a total of 8 h. The isolate had been obtained from the spleen of a mouse surviving IP challenge with 1106 s and euthanized on day 14. The viable counts recovered from the isolate-infected cells were greater than those of the parent strain at all three time points (p ≤ 0.0013) (TIFF 133 kb)
