# Supplementary material for: Characterization of in vitro phenotypes of Burkholderia pseudomallei and Burkholderia mallei strains potentially associated with persistent infection in mice
Source: Arch Microbiol. 2016 Oct 13;199(2):277–301. doi: 10.1007/s00203-016-1303-8 (PMC5306356; doi:10.1007/s00203-016-1303-8)
Supplement: Supplementary file 1 — Supplementary material 1 (DOCX 17 kb) [file 203_2016_1303_MOESM1_ESM.docx]

| **Supplementary Table 1**. Sources of *B*. *mallei* and *B*. *pseudomallei* strains | | | | |  |
| --- | --- | --- | --- | --- | --- |
| **Strain** | **Country of origin** | | **Collection date*** | **Available history and strain characteristics** | **Reference or source**** |
| *Bm* FMH | U.S. | | 2000 | Laboratory-acquired nonfatal infection with ATCC 23344/China7; | USAMRIID UCC,  Srinivasan 2001 |
|  |  |  |  | virulent in hamsters | Romero 2006 |
| *Bm* Turkey 1 | Turkey | | NK | Attenuated in hamsters | USAMRIID TC (DeShazer), from USDA (D. Miller, L. Schlater) |
| *Bm* NCTC 10299 | Burma | | 1944 | human clinical isolate; virulent in hamsters | USAMRIID TC; DeShazer, unpub. |
|  | | |  |  |  |
| *Bp* 316c | | Thailand | 1986–1989 | blood, human case, ceftazidime R | USAMRIID TC; Dance 1991 |
| *Bp* 406e | | Thailand | 1988 | toe swab, systemic human case | USAMRIID UCC, DeShazer 1997 |
| *Bp* 1026b | | Thailand | 1993 | blood sample, nonfatal human case | USAMRIID UCC, DeShazer 1999 |
| *Bp* 1106a | | Thailand | 1993 | human case, protracted, developed liver abscess | USAMRIID UCC, Tuanyok 2008 |
| *Bp* NCTC 4845 | | Singapore | 1935 | monkey isolate | USAMRIID TC, Mack 1998 |
| *Bp* K96243 | | Thailand | 1996 | fatal human case sample/diabetic | USAMRIID UCC, Holden 2004 |
| *Bp* HBPUB10134a | | Thailand | 2010 | human case, tracheal aspirate | USAMRIID UCC |
| *Bp* MSHR305 | | Australia | 1994 | fatal human case, encephalomyelitis | USAMRIID UCC, Tuanyok 2008 |
| *Bp* MSHR668 | | Australia | 1995 | blood, nonfatal human case, encephalomyelitis | USAMRIID UCC, Tuanyok 2008 |
| *Bp* MSHR5855 | | Australia | 2011 | Sputum, suspected inhalational human case | USAMRIID UCC, original source Royal Darwin Hosp. Diag. Lab. |
| *Bp* Bp22 | | Singapore | 1989 | fatal human case, pneumonia/sepsis; highly virulent in mice | DSO National Laboratories (Singapore) |
| *NK = not known. **USAMRIID Unified Culture Collection (UCC) and Therapeutics Core (TC) group. | | | | | |
