# Supplementary material for: Characterization of in vitro phenotypes of Burkholderia pseudomallei and Burkholderia mallei strains potentially associated with persistent infection in mice
Source: Arch Microbiol. 2016 Oct 13;199(2):277–301. doi: 10.1007/s00203-016-1303-8 (PMC5306356; doi:10.1007/s00203-016-1303-8)
Supplement: Supplementary file 2 — Supplementary material 2 (DOCX 18 kb) [file 203_2016_1303_MOESM2_ESM.docx]

| **Supplementary Table 2**. Chemical sensitivity screening with Biolog panel GENIII: *Burkholderia* prototype strains | | | | | | | |
| --- | --- | --- | --- | --- | --- | --- | --- |
|  |  | **Sensitivity (OD_630_)^*^** | | | | | |
|  |  | ***B*. *mallei* 23344 (China7)** | | | ***B*. *pseudomallei* K96243** | | |
| **Well** | **Chemical test** | **Mean** | **SD** | **n** | **Mean** | **SD** | **n** |
| A10 | Pos. ctrl. | 0.876 | 0.105 | 4 | 1.644 | 0.141 | 4 |
| B10 | 1% NaCl | 0.699 | 0.140 | 4 | 1.279 | 0.153 | 4 |
| C10 | sodium lactate | 1.006 | 0.155 | 4 | 1.841 | 0.167 | 4 |
| D10 | troleandomycin | **-0.032** | 0.049 | 4 | 1.356 | 0.309 | 4 |
| E10 | lincomycin | 0.499 | 0.080 | 4 | 1.636 | 0.145 | 4 |
| F10 | vancomycin | 0.872 | 0.107 | 4 | 1.730 | 0.181 | 4 |
| G10 | nalidixic acid | **0.332** | 0.244 | 4 | 1.204 | 0.233 | 4 |
| H10 | aztreonam | **0.227** | 0.056 | 4 | **0.097** | 0.040 | 4 |
| A11 | pH 6 | 0.805 | 0.195 | 4 | 1.734 | 0.109 | 4 |
| B11 | 4% NaCl | **-0.076** | 0.034 | 4 | **-0.063** | 0.066 | 4 |
| C11 | fusidic acid | **-0.035** | 0.035 | 4 | **0.024** | 0.063 | 4 |
| D11 | rifampin | 0.921 | 0.207 | 4 | 1.667 | 0.187 | 4 |
| E11 | guanidine HCl | **-0.019** | 0.066 | 4 | **0.155** | 0.056 | 4 |
| F11 | tetrazolium violet | 1.207 | 0.487 | 4 | 2.908 | 0.022 | 4 |
| G11 | LiCl | **-0.056** | 0.047 | 4 | **-0.022** | 0.151 | 4 |
| H11 | sodium butyrate | **0.015** | 0.084 | 4 | **0.074** | 0.119 | 4 |
| A12 | pH 5 | 0.688 | 0.257 | 4 | 1.828 | 0.152 | 4 |
| B12 | 8% NaCl | **-0.081** | 0.036 | 4 | **-0.141** | 0.015 | 4 |
| C12 | D-serine | **-0.054** | 0.040 | 4 | **-0.072** | 0.028 | 4 |
| D12 | minocycline | **-0.049** | 0.043 | 4 | 1.267 | 0.252 | 4 |
| E12 | niaproof 4 | **-0.076** | 0.033 | 4 | **0.481** | 0.073 | 4 |
| F12 | tetrazolium blue | 1.686 | 0.447 | 4 | 3.023 | 0.049 | 4 |
| G12 | potassium tellurite | **-0.034** | 0.030 | 4 | 0.877**^**^** | 0.971 | 4 |
| H12 | sodium bromate | **-0.058** | 0.032 | 4 | **-0.121** | 0.017 | 4 |
|  | | | | | | | |

*The criteria for sensitivity are similar to those defined by Biolog: **Bolded** = Growth densities (OD_630_) less than half that of positive control well A10 are sensitive.

No change = Growth densities similar to A10 are resistant.

**Density compared to A10 was variable (different experiments).
