# Supplementary material for: Characterization of in vitro phenotypes of Burkholderia pseudomallei and Burkholderia mallei strains potentially associated with persistent infection in mice
Source: Arch Microbiol. 2016 Oct 13;199(2):277–301. doi: 10.1007/s00203-016-1303-8 (PMC5306356; doi:10.1007/s00203-016-1303-8)
Supplement: Supplementary file 3 — Supplementary material 3 (DOCX 23 kb) [file 203_2016_1303_MOESM3_ESM.docx]

| **Supplementary Table 3**: *In vitro* phenotyping screening of *in vivo* isolates: *B. pseudomallei* strain K96243 | | | | | | | | | | | | | | | |  |
| --- | --- | --- | --- | --- | --- | --- | --- | --- | --- | --- | --- | --- | --- | --- | --- | --- |
|  |  |  | **Specific chemical sensitivities^c^** | | | | | | | | | | | | | |
| **Strain^a^** | **Biolog - No. Resistant/ total(23)^b^** | **Change** | **paraquat 2.5 uM** | **NaCl: 1%, 4%** | **nalidixic acid: 5, 50 µg/ml** | **niaproof4: 0.027, 0.10%** | **RNI: 2 mM** | **pH5** | **CecropinA** | **LL37** | **masto-paran7** | **magainin** | **melittin** | **Ca-Ma** | **BMAP-18** | **bactenicin** |
| K96243 parent^d^ | 11^e^ | - | S | S, S+ | R/S, S+ | S, S+ | S+ | R | R/S | nd | S | S | S | S | nd | nd |
| 70-1 | 12 | **R**-tellurite | NC^f^ | NC | NC | NC | NC | NC | NC | nd | NC | NC | NC | NC | nd | nd |
| 70-2, -3 | 12 | **R-**niaproof |  |  |  |  |  |  |  |  |  |  |  |  |  |  |
| K96243 parent | nd | - | S | S, S+ | R/S, S+ | S, S+ | S+ | R | R | R | R/S | R | R | R | R | R/S |
| 70-1 | nd | - | nd^f^ | nd | nd | nd | nd | nd | NC | NC | **R** | NC | NC | NC | NC | NC |
| 70-2 | nd | - | S | **R/S**, S+ | R, S+ | S, S+ | S+ | NC | NC | NC | NC | NC | R/S | R/S | NC | NC |
| K96243parent | 11-12 | - | nd^f^ | nd | nd | nd | nd | nd | R | R/S | S | R | R/S | R/S | S | R |
| 70-1 | 13-14 | S-fus.acid; **R/S**-niaproof, **R**-tellurite | nd | nd | nd | nd | nd | nd | NC | **R** | **R** | NC | **R** | NC | **R** | NC |
| 70-2 | 11 | S-1%Nacl, fus.acid.  **R-**niaproof | nd | nd | nd | nd | nd | nd | NC | **R** | **R/S** | NC | NC | NC | NC | NC |
| 70-3 | 12 | S-fus.acid;  **R**-niaproof | nd | nd | nd | nd | nd | nd | NC | **R** | NC | NC | **R** | **R** | **R** | NC |
| ^a^With the exception of the infecting strain (parent), the strains are identified by the day post-inoculation on which spleens were collected from infected mice (day 70), and the isolate number.  Responses of the isolates to the antimicrobials which varied from the parent are **bolded** (more resistant) or grey (more sensitive).  ^b^Sensitivities were determined with the Gen III panel of 23 chemicals (Biolog). The Biolog criteria were used for resistance (R), > 50% positive control OD_630_, sensitive (S), <50% pos. control, and borderline (R/S).  ^c^Sensitivities to specific chemicals and antimicrobial peptides were determined by OD_630_ readings: values >75% positive control (resistant - R), values >50%, <75% pos. ctrl (borderline - R/S), < 50% pos. ctrl (Sensitive - S), and <2x negative control (highly sensitive - S+).  ^d^Strain K96243 varies in chemical sensitivities depending on the source of the strain; it also exhibits inter-experimental variability (same strain). Responses to several chemicals in the Biolog system showed this variability (footnote e). Thus the parent strain was included in all experiments for comparison with the isolates; results of several assays are shown.  ^e^In Biolog experiments, strain K96243 was resistant to 11 – 13 conditions: 1% NaCl, Na lactate, pH5 and 6, tetrazolium blue and purple, vancomycin, rifampin, lincomycin, troleandomycin, and minocycline. It was usually sensitive to 8-10 conditions: NaCl (4%, 8%), fusidic acid, D-serine, guanidine HCl, LiCl, Na butyrate, Na Bromate. It was variable to niaproof4, K tellurite, aztreonam, nalidixic acid, and sometimes fusidic acid. In the individual chemical sensitivity assays, K96243 was generally highly sensitive (S+) to 4% NaCl, S to 1% NaCl, S to low niaproof 4 (0.11%) and R to 0.03%; it was R to low (5 µg/ml) and S+ to high (50 µg/ml) nalidixic acid.  ^f^NC = same as K96243 parent (no change). nd = not done | | | | | | | | | | | | | | | | |
