# Supplementary material for: Characterization of in vitro phenotypes of Burkholderia pseudomallei and Burkholderia mallei strains potentially associated with persistent infection in mice
Source: Arch Microbiol. 2016 Oct 13;199(2):277–301. doi: 10.1007/s00203-016-1303-8 (PMC5306356; doi:10.1007/s00203-016-1303-8)
Supplement: Supplementary file 4 — Supplementary material 4 (DOCX 23 kb) [file 203_2016_1303_MOESM4_ESM.docx]

| **SupplementaryTable 4.** *In vitro* phenotype screening of *B. pseudomallei* strain 1026b and in vivo isolates | | | | | | | | | | | | | | | | | |
| --- | --- | --- | --- | --- | --- | --- | --- | --- | --- | --- | --- | --- | --- | --- | --- | --- | --- |
|  | **Biolog -** |  | **Specific chemical sensitivities^c^** | | | | | | | | | | | | | | |
| **Strain^a^** | **No. Resistant/ total (23)^b^** | **Change** | **paraquat 2.5 uM** | **NaCl: 1%, 4%** | | **nalidixic acid: 5, 50 µg/ml** | **niaproof 4: 0.027, 0.10%** | **RNI: 2 mM** | **pH5** | **cecropin A** | **LL37** | **masto-paran7** | **magainin** | **melittin** | **CA-MA** | **BMAP-18** | **bacten-ecin** |
| ***In vivo* isolates: Day 30 Aerosol** | | |  |  |  | |  |  |  |  |  |  |  |  |  |  |  |
| 1026b | 13^d^ | R-tell, guanHCL | S | S, S+ | R, S+ | | S, S+ | S+ | R | R | R | R | R | R | R | R/S | R |
| 30-1 | 13 | NC^e^ | NC | NC | NC | | NC | NC | NC | NC | NC | NC | NC | NC | NC | NC | NC |
| 30-2 | 13 | NC | NC | NC | NC | | NC | NC | NC | NC | NC | NC | NC | NC | NC | NC | NC |
|  |  |  |  |  |  | |  |  |  |  |  |  |  |  |  |  |  |
| 1026b | 13^d^ | R/S-niaproof,  R-tell., guanHCL | S | R/S, S+ | R, S+ | | S, S+ | S+ | R | R | R | R | R | R | R | R/S | R/S |
| 30-3 | 13 | S-niaproof | NC | NC | NC | | NC | NC | NC | NC | NC | NC | NC | NC | NC | NC | NC |
| ***In vivo* isolates: Day 60 IP** | | |  |  |  | |  |  |  |  |  |  |  |  |  |  |  |
| 1026b | 11^d^ | S-tellurite, niaproof, guanHCL | S | S, S+ | S, S+ | | S, S+ | S+ | R | S | R/S | S | R | S | S | S | R/S |
| 60-1 | 11 | NC | NC | NC | **R**, S+ | | NC | NC | NC | NC | NC | NC | NC | NC | NC | S | S |
| 60-2 | 11 | NC | NC | NC | **R/S**, S+ | | NC | NC | NC | NC | NC | NC | R/S | NC | **R/S** | **R** | NC |
| 60-3 | 11 | NC | NC | NC | **R/S**, S+ | | NC | NC | NC | NC | NC | **R/S** | NC | NC | NC | **R/S** | S |
|  |  |  |  |  |  | |  |  |  |  |  |  |  |  |  |  |  |
| 1026b | 12^d^ | R-tellurite | S | S, S+ | R/S, S+ | | S, S+ | S+ | R | R/S | R | R | R | R/S | R | S | R/S |
| 60-1 | 12 | NC | NC | NC | **R**, S+ | | NC | NC | NC | NC | NC | NC | NC | NC | NC | NC | **R** |
| 60-2 | 12 | NC | NC | NC | NC | | NC | **S** | NC | NC | NC | NC | NC | NC | NC | NC | **R** |
| 60-3 | 11 | R/S-1%NaCl | NC | NC | NC | | NC | **S** | NC | NC | NC | NC | NC | NC | NC | **R/S** | **R** |
| ^a^With the exception of the infecting strain (parent 1026b), the strains are identified by the day post-inoculation on which spleens were collected from infected mice (day 30 or 60), and the isolate number. Responses of the isolates to the antimicrobials which varied from the parent are **bolded** (more resistant) or grey (more sensitive).  ^b^Sensitivities were determined with the GEN III panel of 23 chemicals (Biolog). The Biolog criteria were used for resistance (R), >50% positive control OD_630_, sensitive (S), < 50% pos. control, and borderline (R/S).  ^c^Sensitivities to specific chemicals and antimicrobial peptides are determined by OD_630_ readings: values >75% positive control (resistant- R), values >50%, <75% pos. ctrl (borderline-R/S), <50% pos. ctrl (sensitive-S), and <2x negative control (highly sensitive-S+).  ^d^In many Biolog experiments, *Bp* strain 1026b was resistant to 11 conditions: 1% NaCl, Na lactate, pH5 and 6, tetrazolium blue and purple, vancomycin, rifampin, lincomycin, troleandomycin, and nalidixic acid. It was sensitive to 12 conditions: 4% and 8% NaCl, fusidic acid, D-serine, minocycline, guanidine HCL, niaproof, potassium tellurite, LiCL, Na butyrate, Na bromate, and aztreonam. However 1026b was often variably resistant or sensitive to K tellurite, niaproof-4, and occasionally guanidine HCL; inter-experimental variability in sensitivities to these chemicals was typical of *B. pseudomallei* strains.  ^e^NC = same as 1026b parent (no change). | | | | | | | | | | | | | | | | | |
