# Supplementary material for: Characterization of in vitro phenotypes of Burkholderia pseudomallei and Burkholderia mallei strains potentially associated with persistent infection in mice
Source: Arch Microbiol. 2016 Oct 13;199(2):277–301. doi: 10.1007/s00203-016-1303-8 (PMC5306356; doi:10.1007/s00203-016-1303-8)
Supplement: Supplementary file 5 — Supplementary material 5 (DOCX 26 kb) [file 203_2016_1303_MOESM5_ESM.docx]

| **Supplementary Table 5 A**. Colony morphology of in vitro isolates of *B. pseudomallei* 406e | | | | | | | | | | | | | | |
| --- | --- | --- | --- | --- | --- | --- | --- | --- | --- | --- | --- | --- | --- | --- |
|  |  |  |  |  |  |  |  | |  | |  |  | |  |
| **Strain** | **Characteristic:** | **1- SBAP** |  | **2- GTA** |  | **3- Ashdowns** |  | | **4- OF-PBL** | | **5- PC/BCA** | **6- Skim milk  zone (mm)** | |  |
|  | | majority | variant | majority | variant | majority | variant | |  | |  |  | |  |
| Bp 406e  parent | 1- size | 1-1.5 mm | 3.5 mm | 1.5 mm | 3.5 mm | 1 mm | 2 mm | | 1.5 mm | | 1 mm | 4 mm | |  |
|  | 2- color of colony | greyish-white | NC | cream | NC | light purple | dark purple | | lt yellowish | | pale pinkish grey |  | |  |
|  | 3-translucency/ opacity | opaque | NC | opaque | NC | opaque | opaque ctr, translucent edge | | opaque | | opaque |  | |  |
|  | 4- moistness | moist, not shiny | shiny, moist | dry; moister  in CG area | shiny, moist | moist | shiny, moist | | shiny, moist | | shiny, moist |  | |  |
|  | 5-circumference shape | uniform, round | NC | uniform, round | NC | uniform, round | uniform, round | | round | | round |  | |  |
|  | 6-surface texture/shape | smooth, sl umbon. ctr | smooth,convex | smooth, sl umbon. ctr | smooth,convex | smooth (small) | smooth, convex to sl umbon. | | smooth, shiny,sl  convex | | smooth, shiny,sl convex |  | |  |
|  | 7-agar color change | none to sl greening | NC | NA | NC | NA | NA | | extensive - yellow | | pink |  | |  |
|  |  |  |  |  |  |  |  | |  | |  |  | |  |
| ***IN VIVO* ISOLATES:** Differences compared to BURK 168 parent - | | | | | | | | | | | | | | |
|  |  |  |  |  |  |  |  |  | |  | | |  | |
| 17-1 | 1 | 3 - 3.5 mm | NONE | NC | NONE | NC | NONE | ~0.5 mm | | 2 mm | | | 5 mm | |
|  | 2 | NC |  | " |  | " |  | NC | |  | | |  | |
|  | 3 | opaque edge, trans. ctr-half colony each |  | " |  | " |  | " | |  | | |  | |
|  | 4 | NC |  | " |  | " |  | " | |  | | |  | |
|  | 5 | sl umbonated ctr |  | " |  | " |  | " | |  | | |  | |
|  | 6 | NC |  | " |  | " |  | " | |  | | |  | |
|  | 7 | NC |  | " |  | " |  | " | |  | | |  | |
|  |  |  |  |  |  |  |  |  | |  | | |  | |
| 17-2 | 1 | 3 mm (>crowded) | NONE | NC | NONE | NC | NONE | NC | | NC | | | 4 mm | |
|  | 2 | NC |  | " |  | " |  | " | | " | | |  | |
|  | 3 | opaque edge, trans. ctr-half colony each |  | " |  | " |  | " | | " | | |  | |
|  | 4 | NC |  | " |  | " |  | " | | " | | |  | |
|  | 5 | sl umbonated ctr |  | " |  | " |  | " | | " | | |  | |
|  | 6 | NC |  | " |  | " |  | " | | " | | |  | |
|  | 7 | NC |  | " |  | " |  | " | | " | | |  | |

| **Supplementary Table 5 B**. Colony morphologies of *in vivo* isolates of *B*. *pseudomallei* 406e | | | | | | | | | | |
| --- | --- | --- | --- | --- | --- | --- | --- | --- | --- | --- |
| **Strain** | **Characteristic*** | | | **1- SBAP** |  | **2- GTA** | **3- Ashdowns** | **4- OF-PBL** | **5- PC/BCA** | **6- Skim milk zone (mm)** |
|  |  | | |  | variant |  |  |  |  |  |
| 406e parent | 1. size | | | 2.5–3 mm | - | 3 mm | 1.5–2 mm | 1 mm | 1.5–2 mm | 6 mm |
|  | 2. color of colony | | | greyish white | - | Cream white | medium purple | lt yellowish | pale pinkish grey |  |
|  | 3. translucency/opacity | | | opaque | - | opaque | opaque | opaque | opaque |  |
|  | 4. moistness | | | moist, not shiny | shiny, moist | dry; moister CG | dry; moister CG | shiny, moist | shiny, sl moist |  |
|  | 5. circumference shape | | | uniform, round, regular | - | uniform, round, regular | round, regular | round | round, regular, uniform |  |
|  | 6. surface texture/shape | | | sl rough texture, smooth edge, sl umbon ctr | smooth, convex | flat, rough, regular | rough, flat, sl umbon, typical | smooth, shiny, flat to sl raised | smooth, flat to sl raised |  |
|  | 7. agar color change | | | none | - | NA | NA | yellow | pink |  |
| ***IN VIVO* ISOLATES: DAY 34**** | | | | | | | | | | |
| 34-1 | | 1 | sl larger, 3–3.5mm | | None: | NC** | NC | sl larger,  1.5 mm | larger, 3–4 mm | 5 mm |
|  | | 2 | NC | | one type only | " | " | NC | pinkish white |  |
|  | | 3 | NC | |  | " | " | " | NC |  |
|  | | 4 | moister, shinier | |  | " | " | " | less moist |  |
|  | | 5 | NC | |  | " | " | " | NC |  |
|  | | 6 | NC | |  | " | " | " | concave or sl umbon,  rough ctr, smooth edge |  |
|  | | 7 | NC | |  | " | " | " | NC |  |
| 34-2 | | 1 | same as 34-1 | | None | NC | NC | NC | same as 34-1 | 4 mm |
|  | | 2 | " | |  | " | " | " | " |  |
|  | | 3–7 | " | |  | " | " | " | " |  |
| 34-3 | | 1 | same as 34-1 | | None | NC | NC | same as 34-1 | same as 34-1 | 4 mm |
|  | | 2 | " | |  | " | " | " | " |  |
|  | | 3–7 | " | |  | " | " | " | " |  |
| 34-4 | | 1 | NC | | None | NC | NC | same as 34-1 | same as 34-1 | 3–3.5 mm |
|  | | 2 | " | |  | " | " | " | " |  |
|  | | 3–7 | " | |  | " | " | " | " |  |
| 34-5 | | 1 | NC | | None | NC | NC | NC | same as 34-1 | 4 mm |
|  | | 2 | " | |  | " | " | " | " |  |
|  | | 3–7 | " | |  | " | " | " | " |  |
| *Abbreviations: CG = confluent growth; sl = slightly; umbon = umbonated; ctr = center; lt = light.  **Differences compared to *Bp* 406e parent are shown. NC = no change (same as parent). | | | | | | | | | | |
