# Supplementary material for: Characterization of in vitro phenotypes of Burkholderia pseudomallei and Burkholderia mallei strains potentially associated with persistent infection in mice
Source: Arch Microbiol. 2016 Oct 13;199(2):277–301. doi: 10.1007/s00203-016-1303-8 (PMC5306356; doi:10.1007/s00203-016-1303-8)
Supplement: Supplementary file 6 — Supplementary material 6 (DOCX 17 kb) [file 203_2016_1303_MOESM6_ESM.docx]

| **Supplementary Table 6**. *B*. *pseudomallei* chemical sensitivities of isolates by chemical | | |
| --- | --- | --- |
|  | **Strains with isolates varying from parent^a^** | |
| **Chemical** | **More resistant** | **More sensitive** |
| niaproof 4 | MSHR668 (Biolog)^c^, 1106a, MSHR305,  406e, K96243^b^ | K96243b, MSHR668 (antimic. sens.)^c^,  1026b (BiOLOG)^c^ |
| nalidixic acid | K96243, 1106a^b^, MSHR305, 406e, 1026b | 1106a^b^ |
| mastoparan 7 | K96243^b^, 1106a, MSHR305, 1026b^b^ | K96243^b^ |
| BMAP-18 | K96243, 406e, 1026b |  |
| minocycline | MSHR668^b^, MSHR305 | MSHR668^b^, K96243^c^ |
| nitrite | MSHR305 | K96243, 1106a |
| NaCl | K96243, 1106a, 406e |  |
| K tellurite | MSHR668^b^, 1106a, MSHR305 | MSHR668^b^ |
| magainin | 1106a, MSHR305 |  |
| D-serine | 1106a | 406e |
| melittin | K96243 | 406e |
| others:^d^ |  |  |
| guanidine HCl | MSHR668 |  |
| aztreonam | 1106a |  |
| LL-37 | K96243 |  |
| CA-MA | 1026b |  |
| bactenecin | 1026b | 1026b |
| ^a^Spleen isolates from aerosol or IP challenge experiments of seven *Bp* strains were obtained (17 total experiments). The variant responses of the isolates consisted of significant increases in either sensitivity (S) or resistance (R) to the antimicrobial chemical compared to the challenge strain, as described in the methods. The data do not include isolates exhibiting no differences from the parent. For strain HBPUB10134a, no isolates were obtained from survivor spleens cultured later than 14 days after challenge.  ^b^The sensitivity of isolates collected at early and later time points differed (nalidixic acid, niaproof 4, mastoparan 7, and K tellurite) or individual isolates from a given challenge experiment responded differently, being either more sensitive or more resistant than the parent (minocycline, niaproof 4, potassium tellurite and mastoparen 7) .  ^c^Different responses by the same isolate were observed in Biolog and individual antimicrobial sensitivity microtiter assays; but chemical concentrations in the Biolog system are proprietary and unavailable.  ^d^For one *Bp* strain each, the chemical sensitivity of an isolate(s) varied from that of the parent as follows: aztreonam (R), , guanidine HCl (R), LL-37 (R ), CA-MA (R), and bactenecin (variable). Peptides BMAP-18, CA-MA, bactenecin, and LL-37 were tested in four of the six strains only. | | |
