# Supplementary material for: Characterization of in vitro phenotypes of Burkholderia pseudomallei and Burkholderia mallei strains potentially associated with persistent infection in mice
Source: Arch Microbiol. 2016 Oct 13;199(2):277–301. doi: 10.1007/s00203-016-1303-8 (PMC5306356; doi:10.1007/s00203-016-1303-8)
Supplement: Supplementary file 7 — Supplementary material 7 (DOCX 19 kb) [file 203_2016_1303_MOESM7_ESM.docx]

| **Supplementary Table 7**. Phenotypes of macrophages infected with *B. pseudomallei* parent strain and spleen isolates: Bacterial survival | | | | | | | | | |
| --- | --- | --- | --- | --- | --- | --- | --- | --- | --- |
|  |  |  | **Adherence and phagocytosis^c^** | | | **Recovery - final sample^d^** | | | |
| **Strain^a^** | **Panel^b^** | **MOI** | **Inoculum (CFU/well)** | **Cell-associated (1 h) % inoculum** | **Phagocytosed (3 h) % inoculum** | **Macrophage infection time (h)** | **No. CFU/well** | **Compared to inoculum (%)** | **Compared to 3 h counts (%)** |
| K96243 |  | 13.3 | 1.33 x 10^7^ | 1.70 | 0.72 | 8.0 | 9.03x10^5^ | 6.8 | 941 |
| Bp 70-1 | A | 11.0 | 1.10 x 10^7^ | 3.70 | 2.20 | " | 1.60x10^6^ | 14.5 | 667^e^ |
| MSHR668- expt#1 |  | 12.2 | 9.00 x 10^6^ | nd^f^ | nd^f^ | 7.0 | 1.67x10^6^ | 18.6 | NA^g^ |
| Bp 41-1 | B | 11.2 | 8.30 x 10^6^ | " | " | " | 6.11x10^6^ | 73.4 | " |
| Bp 41-4 | inset | 13.1 | 9.70 x 10^6^ | " | " | " | 3.61x10^6^ | 37.2 | " |
| MSHR668- expt#2 |  | 38.5 | 1.5 x 10^7^ | 1.15 | 0.46 | 8.0 | 5.08x10^5^ | 3.4 | 735 |
| Bp 41-1 | B | 32.3 | 1.26 x 10^7^ | 1.17 | 0.61 | " | 9.02x10^5^ | 7.2 | 1183 |
| 1106a |  | 12.3 | 6.75 x 10^6^ | 2.1 | 0.63 | 8.0 | 9.82x10^4^ | 1.4 | 231 |
| Bp 62-3 | C | 14.1 | 7.75 x 10^6^ | 6.1 | 1.20 | " | 1.85x10^5^ | 2.4 | 199^e^ |
| 1026b |  | 9.5 | 0.90 x 10^7^ | 1.87 | 1.34 | 8.0 | 8.9x10^5^ | 9.9 | 742 |
| Bp 60-3 | D | 11.4 | 1.08 x 10^7^ | 1.06 | 0.55 | " | 1.10x10^6^ | 10.2 | 1839 |
| MSHR305 |  | 15.4 | 1.35 x 10^7^ | nd^f^ | nd^f^ | 6.5 | 2.34x10^5^ | 1.7 | NA^g^ |
| Bp 33-2 | E | 17.5 | 1.54 x 10^7^ | " | " | " | 3.9x10^5^ | 2.5 | " |
| 406e |  | 17.1 | 1.68 x 10^7^ | 1.50 | 1.26 | 8.0 | 9.34x10^5^ | 5.6 | 443 |
| Bp 60-4 | F | 15.5 | 1.52E+07 | 2.30 | 1.80 | " | 8.84x10^5^ | 5.8 | 324 |
| ^a^Indicates the parent strain of *Bp* and an isolate from infected spleen (designated by day post-challenge of necropsy and spleen colony number).  ^b^Fig. 3, panels A-F.  ^c^J774.A1 cells were inoculated with a *Bp* strain at an MOI of 10–20 and incubated for 1 h. The data shown are the mean viable counts recovered from cell lysates (triplicate wells), expressed as a percentage of the inoculum; viable counts were obtained after the 1 h uptake and after incubation of the infected cells in the presence of kanamycin for 2 h to remove unphagocytosed bacteria (3 h total).  ^d^Viable counts recovered after a total of 8 h incubation are shown as the mean no. CFU/well (triplicate wells) and the mean expressed as a percentage of the inoculum or as percentage of the mean value obtained after 3 h.  ^e^Due to the larger % cell loss, the relative no. CFU/well recovered may be underestimated.  ^f^nd = not done.  ^g^NA = not applicable. | | | | | | | | | |
