# Supplementary figures and images for: Characterization of in vitro phenotypes of Burkholderia pseudomallei and Burkholderia mallei strains potentially associated with persistent infection in mice
Source: Arch Microbiol. 2016 Oct 13;199(2):277–301. doi: 10.1007/s00203-016-1303-8 (PMC5306356; doi:10.1007/s00203-016-1303-8)

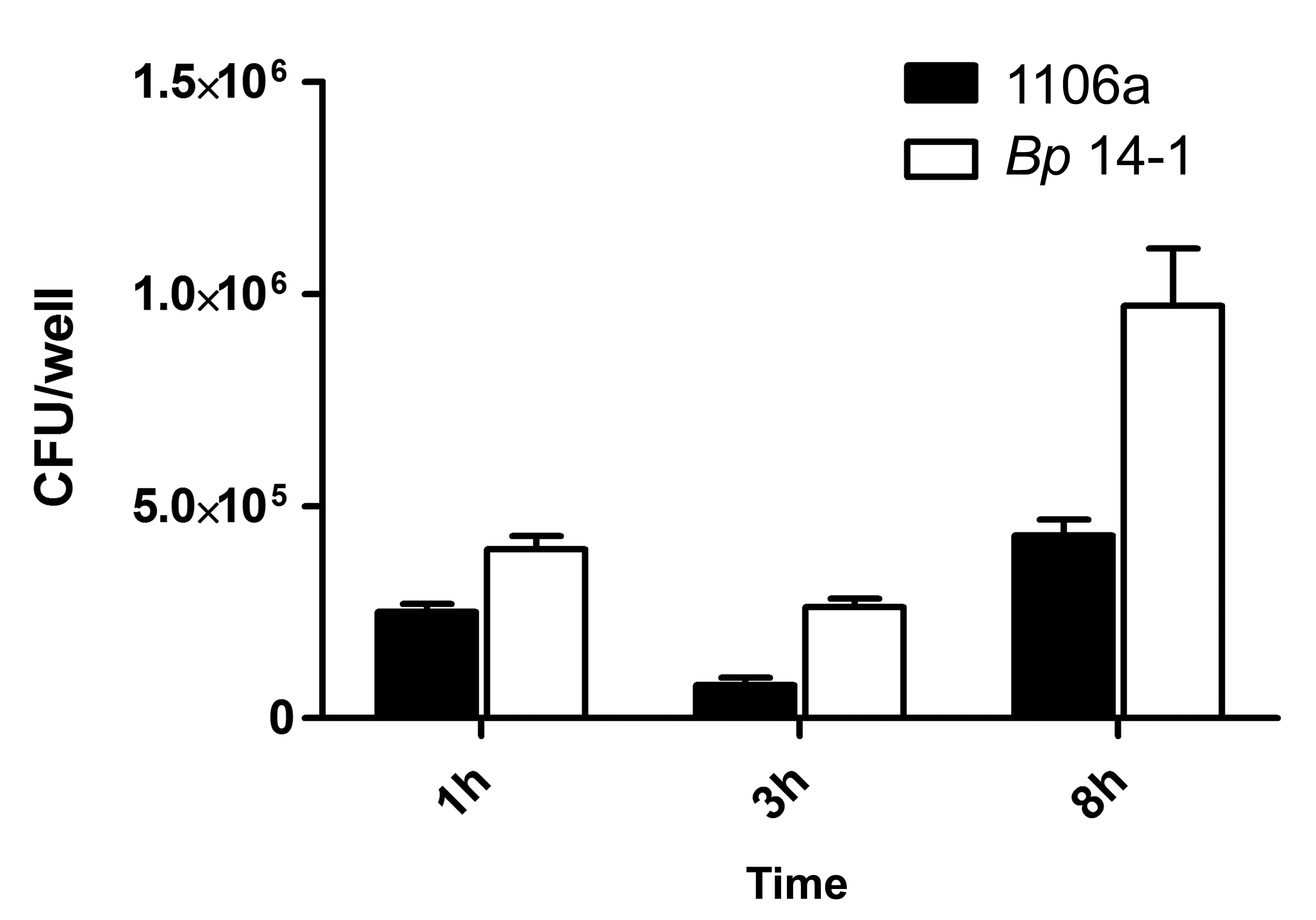

Supplement: Supplementary file 8 — Comparison of macrophage survival of Bp strain 1106a and mouse spleen isolate Bp 14-1. J774.A1 cells were inoculated with MOIs of 16.6 and 11.0, respectively, and incubated for 1 h. The data shown are the mean viable counts (triplicate wells) recovered after the 1-h uptake, incubation of the infected cells in the presence of kanamycin for 2 h (3 h), and after incubation for a total of 8 h. The isolate had been obtained from the spleen of a mouse surviving IP challenge with 1106 s and euthanized on day 14. The viable counts recovered from the isolate-infected cells were greater than those of the parent strain at all three time points (p ≤ 0.0013) (TIFF 133 kb) [file 203_2016_1303_MOESM8_ESM.tif]
